# Cancer Photodynamic Therapy Enabled by Water-Soluble Chlorophyll Protein

**DOI:** 10.1021/acsami.5c01280

**Published:** 2025-03-06

**Authors:** Lixin Liang, Wenjun Wang, Manjia Li, Yingjie Xu, Zhangdi Lu, Jingjing Wei, Ben Zhong Tang, Fei Sun, Rongbiao Tong

**Affiliations:** † Guangxi Key Laboratory of Special Biomedicine, School of Medicine, Guangxi University, Nanning 530004, China; ‡ Department of Chemistry, The Hong Kong University of Science and Technology, Clear Water Bay, Kowloon 999077, Hong Kong, China; § Department of Chemical and Biological Engineering, The Hong Kong University of Science and Technology, Clear Water Bay, Kowloon 999077, Hong Kong, China; ∥ School of Science and Engineering, Shenzhen Institute of Aggregate Science and Technology, The Chinese University of Hong Kong, Shenzhen 518172, Guangdong, China; ⊥ College of Chemical and Environmental Engineering, Anyang Institute of Technology, Anyang 455000, China; # Exponent Ltd., 12 Science Park West Avenue, Unit, Sha Tin 802-803, New Territories, Hong Kong

**Keywords:** chlorophyll, cancer, photodynamic therapy, photosensitizer, water-soluble chlorophyll protein

## Abstract

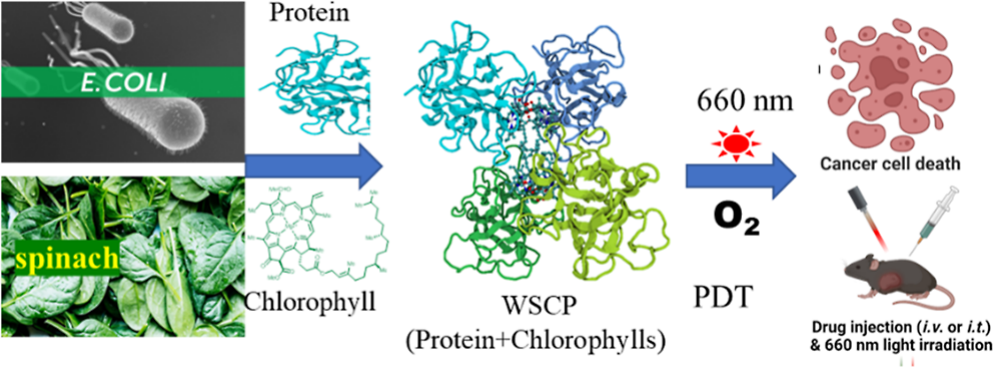

Photodynamic therapy (PDT) has been utilized to treat various malignant cancers for more than a century. However, many photosensitizers (e.g., derivatives of porphyrins, chlorins, etc.) central to PDT are still suffering from limitations such as water insolubility, dark toxicity, photo/thermal-instability, difficult synthesis/preparation, and poor tumor selectivity. Numerous effective strategies include designing new synthetic photosensitizers by exploiting heavy atom effect, aggregation-induced emission effect (AIE), and electronic/energy effects (donor–acceptor, and Förster resonance energy transfer: FRET), and the linkage of activatable and targeting molecules has been developed to address one or more of these limitations. However, these structural modifications of photosensitizing organic molecules are synthetically challenging and unpredictable in terms of efficacy versus toxicity. Herein, we report a new and simple strategy for effective PDT by combining natural spinach-derived chlorophylls (photosensitizer) with natural water-soluble chlorophyll proteins (WSCPs) derived originally from plants and produced heterologously by bacteria (*E. coli*). The recombinant WSCPs (chlorophyll-WSCP) are tetrameric and stable under air/thermal conditions and importantly can produce highly reactive singlet oxygen under red/far-red light irradiation to induce cancer cell death. Our in vivo mouse model studies (melanoma xenografts) further validate the efficacy of the recombinant WSCPs as a new class of water-soluble, nontoxic, and highly efficient photosensitizers for PDT. This work represents the first example of the application of WSCPs in PDT and may advance the clinical applications of PDT for cancer treatment.

## Introduction

**Figure 1:**
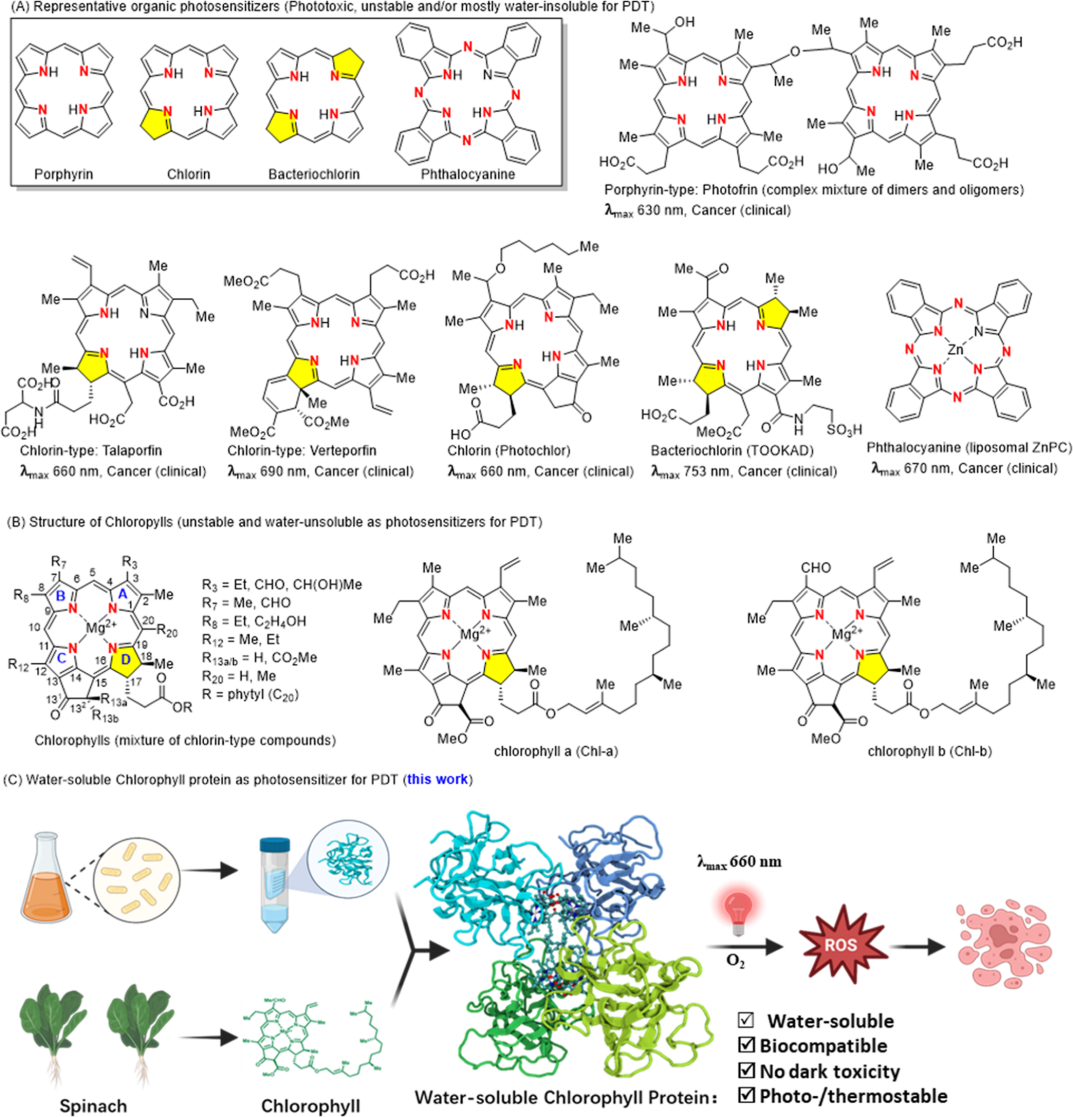
Photosensitizers for photo dynamic therapy (PDT). (A) Structures and limitations of existing organic photosensitizers for PDT. (B) Structure and limitations of chlorophyll as the photosensitizer for PDT. (C) Schematic illustration of preparation and anticancer PDT process of water-soluble chlorophyll protein (WSCP) (PDB ID: 2DRE).

Photodynamic therapy (PDT) has been used for over 100 years as an effective and noninvasive treatment for cancers.^1−4^ It is commonly used for cutaneous tumors, such as precancerous keratosis skin lesions and some nonmelanoma skin cancers,^5,6^ and occasionally for other cancer types including breast, lung, bladder, pancreas, cervix, prostate, and brain.^7^ Compared to conventional treatments (surgery, chemotherapy, and radiotherapy), PDT offers significant clinical advantages such as minimal invasiveness, little drug resistance, and high spatiotemporal precision, which overcome the common limitations of chemo- and/or immuno-therapies.^8,9^ Central to PDT is an effective photosensitizer that upon light irradiation can generate reactive oxygen species (ROS), such as singlet oxygen, to induce cell death.^10−12^ One of the major classes of photosensitizers for tumor destruction^13−19^ is the tetrapyrrole-based macrocyclic molecules such as porphyrins,^20^ chlorins,^21,22^ bacteriochlorins,^21^ or phthalocyanines^23^ (Figure 1A). The hematoporphyrin derivatives (HpDs),^24^ temoporfin (Foscan), verteporfin (Visudyne), and talaporfin (LS11), are representative members of tetrapyrrole-based photosensitizers used clinically with red/far-red light at 630–900 nm, a therapeutic window allowing for deep-tissue penetration.^19^ However, these photosensitizers are not ideal due to their poor water solubility, dark toxicity, and photo/thermal instability, which have hindered their wider use in PDT cancer treatment.^25,26^

Compared to porphyrins (HpD and photofrin) with a weak absorbance at 630 nm (Q-band), chlorin-based photosensitizers (temoporfin and talaporfin) contain a dihydropyrrole (yellow) and can absorb light above 650 nm,^27^ which is advantageous for deeper tissue penetration and faster biodegradation/elimination. This advantage explains why many second-generation photosensitizers are chlorin based.^27−30^ However, these chlorin photosensitizers are still plagued by poor solubility and chemical instability. Naturally occurring chlorins, such as chlorophylls, are nontoxic but insoluble in water and unstable under air conditions (oxidative degradation). Hydrogels and liposomes have been used to deliver chlorophylls with improved water solubility and photostability.^31,32^ Serum albumin^33−35^ has also been explored for delivery of chlorophylls (Ce6) with improved solubility, stability, and biocompatibility. Painstaking chemical modifications^36^ of chlorophylls have been made to address these limitations. However, these strategies for overcoming the intrinsic drawbacks of chlorophylls are not ideal for PDT in clinical applications.^36,37^

Water-soluble chlorophyll proteins (WSCPs) from the *Brassicaceae* family of plants have previously been shown to be remarkably stable photosensitizers capable of generating singlet oxygen in response to red light.^38^ WSCPs form tetramers upon binding to chlorophylls (Figure 1C). Contrary to free chlorophylls, which are insoluble in water and unstable under air/oxygen conditions,^39−42^ the protein-bound chlorophylls are highly soluble in water and extremely stable under harsh conditions (e.g., 100 °C and pH 0–14), being protected by the protein scaffolds from oxidation under light irradiation.^39,43−50^ Hence, we envision that WSCP might be used as a water-soluble, red-light photosensitizer in PDT for cancer treatment while avoiding the trade-off between water solubility and biocompatibility that is often associated with chlorin-based photosensitizers.

In this study, we successfully produced recombinant WSCP using heterologous bacterial expression and in vitro reconstitution with chlorophylls from spinach extracts. The resulting WSCP proved to be an effective photosensitizer for PDT, exhibiting little dark cytotoxicity in vitro but great potency in suppressing tumor growth in mouse models under light illumination.

## Results and Discussion

### Photophysical Properties of WSCPs

**Figure 2:**
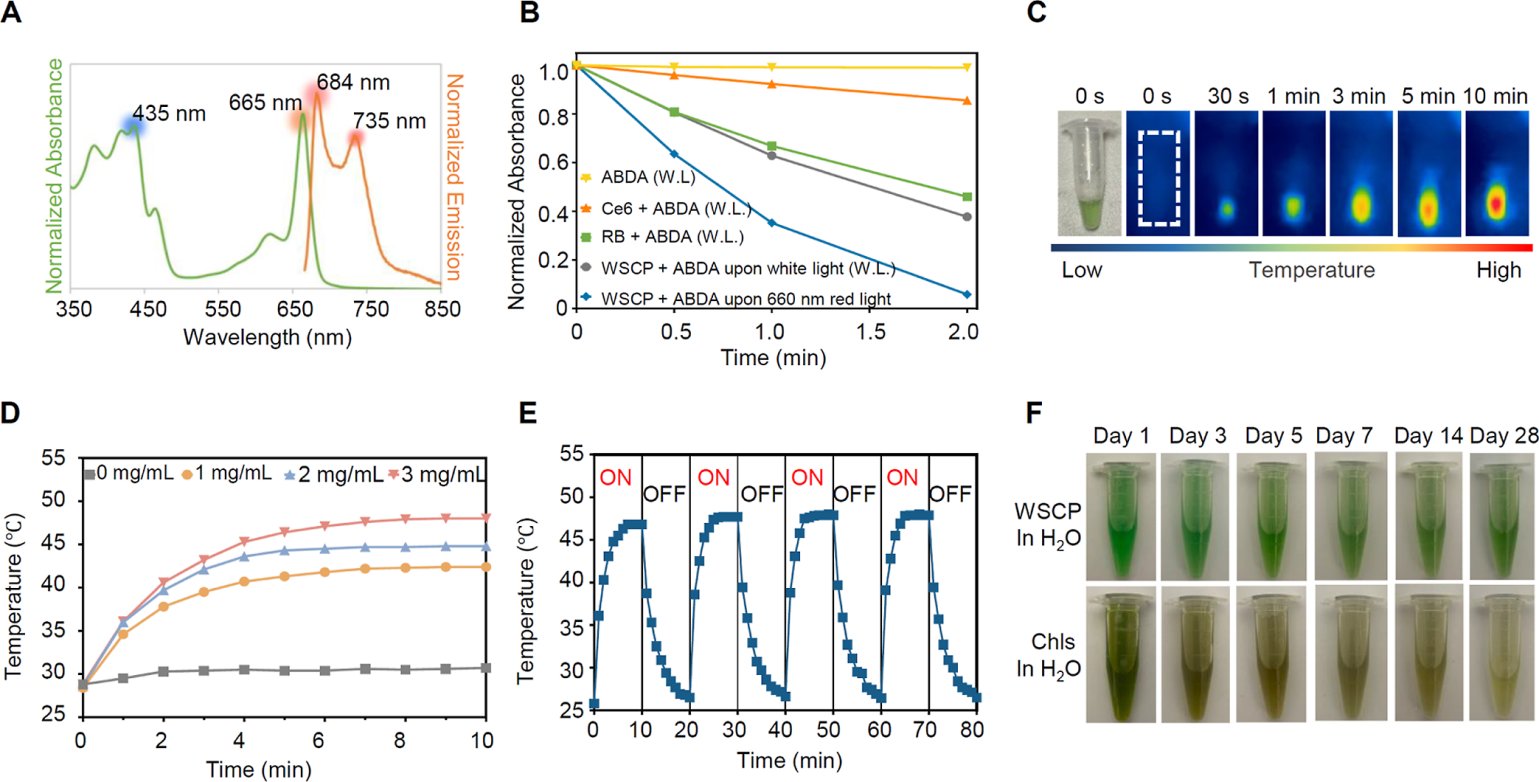
Characterization of the recombinant WSCP. (A) Normalized absorption and emission (excited at 634 nm) spectra of the recombinant WSCP in water. (B) Comparison of WSCP with small-molecule photosensitizers such as Rose Bengal (RB) and Ce6 (porphyrin derivative) in ROS generation. Normalized absorbances (380 nm) of 9,10-anthracenediyl-bis(methylene) dimalonic acid (ABDA) (2 × 10^–5^ M), a singlet oxygen scavenger probe used to detect ROS, upon white-light (W.L.) (1 mW/cm^2^) or red light (600 nm, 7 mW/cm^2^) irradiation in the presence of different photosensitizers (1 × 10^–4^ M) are shown. (C) Photothermal effect of WSCP. Representative infrared thermal images of WSCP (2 mg/mL) upon light irradiation (660 nm, 0.8 W/cm^2^) are shown. (D) Photothermal conversion of WSCP at different concentrations upon light irradiation (660 nm, 0.8 W/cm^2^). Data are presented as mean ± SD (*n* = 4). (E) Photothermal conversion of WSCP (3 mg/mL) over four cycles of light irradiation (660 nm, 0.8 W/cm^2^). (F) Comparison of free and protein-bound chlorophylls in long-term stability under ambient conditions.

WSCPs obtained from plants were reported to absorb light in the range of 350–480 and 600–700 nm.^51^ We successfully produced the recombinant WSCP using heterologous bacterial expression and in vitro reconstitution with chlorophylls from spinach extracts and found that it exhibited the same absorption band in the range of 350–480 and 600–700 nm with maxima at 435 and 665 nm (Figure 2A). The photoluminescence (PL) spectrum of the recombinant WSCP displayed maximal emission peaks at 684 and 735 nm (excited at 634 nm). The absorbance at 665 nm renders WSCP an excellent photosensitizer for potential cancer PDT in deep tissues^52^ with minimal damage to skin and a deeper penetration into tissues.

### Singlet Oxygen Generation by WSCPs

Singlet oxygen generation is key to the therapeutic efficacy of most photosensitizers for PDT. Previous studies from many research groups (Paulsen,^51^ Schmidt,^51^ and Agostini^53^) revealed that WSCP can generate singlet oxygen from molecular oxygen upon irradiation with red light. To confirm the ability of recombinant WSCP for singlet oxygen generation, we used 9,10-anthracenediyl-bis(methylene) dimalonic acid (ABDA) as a singlet oxygen trapping agent to investigate conversion of ground-state oxygen to singlet oxygen. A decrease in absorbance indicated singlet oxygen generation. The efficiency of singlet oxygen generation was compared with that of two commercial photosensitizers, RB and Ce6. As shown in Figure 2B, the absorbance of ABDA decreased significantly from 100% in 2 min under white light irradiation (1 mW/cm^2^) in the presence of RB (46%), Ce6 (86%), and the recombinant WSCP (38%), indicating singlet oxygen generation. Notably, the use of 660 nm red light (7 mW/cm^2^) irradiation led to a substantial decrease in absorbance for recombinant WSCP (100% to 6%), in sharp contrast to the poor efficiency of singlet oxygen generation by the conventional synthetic photosensitizer Ce6 (100% to 76%). Importantly, the recombinant WSCP demonstrated a significantly higher singlet generation efficiency Φ_ROS_ [×10^–3^ s^–1^]^54^ (8.2 under white light irradiation; 24.1 under red light irradiation) compared to RB (6.44 under white light irradiation) and Ce6 (1.3 under white light irradiation; 2.3 under red light irradiation) under the same irradiation conditions (Figure S1 and Table S1). The astonishing efficiency of singlet oxygen generation at 660 nm red light irradiation renders WSCP advantageous to possible PDT application for cancer treatment.

### Photothermal Property

Since the light irradiation typically results in temperature elevation, we evaluated the photothermal effect of the recombinant WSCP. The photothermal effect of WSCP under irradiation was visualized using infrared thermal imaging (Figures 2C and S2). The color of the WSCP solution changed from blue (low temperature) to red (high temperature) over time, indicating that light irradiation of WSCP caused a temperature increase. To further quantify this effect, we measured the temperature (Figure 2D). Under 660 nm red light (0.8 W/cm^2^) irradiation, the temperature of the recombinant WSCP in water increased from ambient temperature (28 °C) to 40–50 °C within 10 min, while the temperature of pure water (control) remained at approximately 29–30 °C. The photothermal effect was positively correlated with concentration: 3 mg/mL of WSCP increased the temperature to nearly 50 °C under light irradiation, while 1 mg/mL of WSCP only elevated the temperature to 40 °C. A cell temperature of 42–50 °C has been reported to cause irreversible cell death or tissue damage (necrosis, microvascular thrombosis, or ischemia); however, this temperature range is insufficient for effective photothermal therapy, which typically requires local temperature elevation above 60 °C.^55^

Nevertheless, these results hinted that the recombinant WSCP, as a photosensitizer, might exert dual light effects: photodynamic and photothermal, in cancer phototherapy. The low-temperature photothermal effect of recombinant WSCP could be potential for low-temperature photothermal therapy, minimizing thermal injury to normal tissues through thermal diffusion, and might be exploited in combination with immune-therapy.^26^ The temperature elevation upon light irradiation prompted us to investigate the photothermal stability of the recombinant WSCP. It is well-known that natural chlorophylls, a green mixture of chlorin-type organic compounds, are extremely unstable under light irradiation, which is a major restriction for PDT. To our delight, the complexation of natural chlorophylls with proteins (WSCP) led to extraordinary stability (Figure 2E). After four cycles of heating (irradiation with red light to 48 °C) and cooling, the photothermal conversion efficiency (16.4% calculated according to reported methods^56^) did not decrease significantly. This is noteworthy because the recombinant WSCP might address the photothermal stability issue, which is one of the common challenges faced by nearly all well-designed organic photosensitizers (porphyrins and chlorins) in PDT.

### Storage Stability

It is not surprising that most synthetic organic photosensitizers are chemically unstable when stored at room temperature without light shielding, which contributes as a minor fraction to the slow progress of PDT in clinical cancer treatment. The tetrameric WSCP complex was reported to be extraordinarily stable (up to 100 °C),^50^ and we investigated the storage stability of the recombinant WSCP in water and in methanol (Figure S3). As shown in Figure 2F, the recombinant WSCP solution remained green with no noticeable degradation after 28 days of storage at ambient temperature under normal room light conditions, while free chlorophylls decomposed within a day. This finding suggests that the water-soluble protein effectively protects chlorophylls from decomposition, and this superior stability could be crucial for the development of the recombinant WSCP as a photosensitizer for PDT.

### Cell Killing Assay of WSCP for PDT

**Figure 3:**
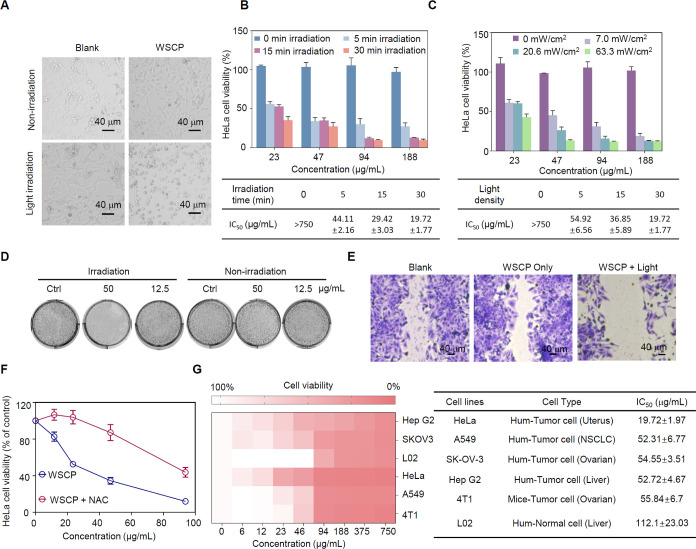
Light-induced cytotoxicity of WSCP against cancerous cells. (A) Representative micrographs showing the influence of WSCP on HeLa cells upon light irradiation (660 nm, 7.0 mW/cm^2^). Scale bar: 40 μm. (B) Influence of light duration (660 nm, 63.3 mW/cm^2^) on cell viability and IC_50_ of WSCP. Data presented as the mean ± SD (*n* = 3). (C) Influence of light intensity (660 nm, 30 min) on cell viability and IC_50_ of WSCP. Data presented as the mean ± SD (*n* = 3). (D) Colony formations of HeLa cells assay were performed to study the long-term effects of recombinant WSCP photoinduced therapy on the survival and proliferation of HeLa cells. In vitro phototherapy with recombinant WSCP inhibited the colony-forming ability of HeLa cells in a concentration-dependent manner. Images are representative of *n* = 3. (E) Recombinant WSCP inhibited the migration of HeLa cells after treatment with irradiation. Scale bar: 40 μm. Images are representative of *n* = 3. (F) Relative viability of HeLa cells after incubation with the recombinant WSCP in the presence or absence of *N*-acetyl-l-cysteine (NAC, a ROS inhibitor) with light irradiation. Data were expressed as the mean ± SD (*n* = 3). (G) In vitro photocytotoxicity of recombinant WSCP toward different cell lines. Data were expressed as the mean ± SD (*n* = 3).

The physicochemical properties (singlet oxygen generation and photo/chemical stability) of WSCP have been reported in the literature and were confirmed by our experiments (Figure 2). However, these properties have not been exploited to develop WSCP as a new class of photosensitizers for PDT. To validate the efficacy of WSCP as a photosensitizer in living cells for PDT, we conducted cell killing experiments using HeLa cells as a model (Figure 3A). HeLa cells were treated with a high concentration of recombinant WSCP (750 μg/mL) in the dark for 24 h and then irradiated with 660 nm red light (7.0 mW/cm^2^) for 15 min. Microscopic visualization revealed that the morphology of HeLa cells changed with condensed cytoplasm and floating, when both WSCP and light were present simultaneously, which demonstrated that the recombinant WSCP was an effective photosensitizer in the tumor cells to generate singlet oxygen upon light illumination. Next, we further quantified the photocytotoxicity of the recombinant WSCP in HeLa cells. HeLa cells were incubated with WSCP in the dark for 24 h and then exposed to 660 nm red light for 5, 15, and 30 min(Figure S4). As shown in Figure 3B, the IC_50_ value was strongly correlated with the light irradiation time: 44.11 μg/mL (5 min), 29.42 μg/mL (15 min), and 19.72 μg/mL (30 min). Without light irradiation, the IC_50_ value was larger than our control limit (>750 μg/mL), indicating extremely low dark-cytotoxicity of WSCP. We also studied the effect of light density (7.0, 20.6, and 63.3 mW/cm^2^) on photocytotoxicity of WSCP (Figures S5 and 3C) and found that higher light density led to greater cytotoxicity: IC_50_ 54.92 μg/mL (7.0 mW/cm^2^), 36.85 μg/mL (20.6 mW/cm^2^), 19.72 μg/mL (63.3 mW/cm^2^), respectively. It is noteworthy that light density had little impact on cell viability when the WSCP concentration was too low (23 μg/mL) or too high (188 μg/mL) (Figure 3C). These results demonstrate that the recombinant WSCP is an efficient photosensitizer under cell culturing conditions for generating singlet oxygen to induce cell death.

### Colony Formation Assay

A cell colony formation assay was performed to assess the antiproliferation effect of the recombinant WSCP as a photosensitizer for PDT on the HeLa (Figure 3D) and A549 (Figure S6) cell lines. Cells were treated with WSCP (50, 25, 12.5, 6, and 3 μg/mL) and irradiated with 660 nm light (31.8 mW/cm^2^ for 15 min) followed by incubation for an additional 14 days in the absence of WSCP. We found that cells treated with 50 μg/mL of WSCP and irradiation lost their proliferation ability (no colony formation), while 25 and 12.5 μg/mL of WSCP and light irradiation substantially inhibited colony formation. Treatments of low concentrations of WSCP (6 and 3 μg/mL) did not result in significant inhibition. These results indicate that photoinduced WSCP therapy can prevent cancer cell proliferation, but its effect was dose dependent. Additionally, the control experiment (with WSCP alone in the absence of light irradiation) confirmed once again that WSCP has no dark toxicity.

### Cell Migration Assay

We also examined the antimigration ability of the recombinant WSCP on HeLa (Figure 3E) and A549 (Figure S7) cells using a scratch wound-healing assay. Comparable cell migration into the scratched areas was observed in the control group (without WSCP) and the group treated with WSCP but without light irradiation. In contrast, the group treated with both WSCP and light irradiation had a larger scratched area, indicating that the recombinant WSCP significantly inhibits the wound-healing and invasion abilities of cancer cells upon light irradiation.

### Effect of ROS Scavenger

Since PDT generates ROS, such as singlet oxygen and hydroxyl radicals, which are lethal to cells, it is expected that ROS scavengers would inhibit ROS reactivity and thus increase cell viability even in the presence of light irradiation. We used *N*-acetyl-l-cysteine (NAC) as an ROS scavenger and examined its effect on HeLa cells treated with recombinant WSCP and red light (Figure 3F). Our experimental results showed that NAC significantly increased cell viability by 20–50% with higher IC_50_ values (27.94–87.65 μg/mL), supporting the conclusion that ROS generated by WSCP upon light irradiation are responsible for its cell-killing ability.

### Cell Killing Assay against Other Cell Lines

**Figure 4:**
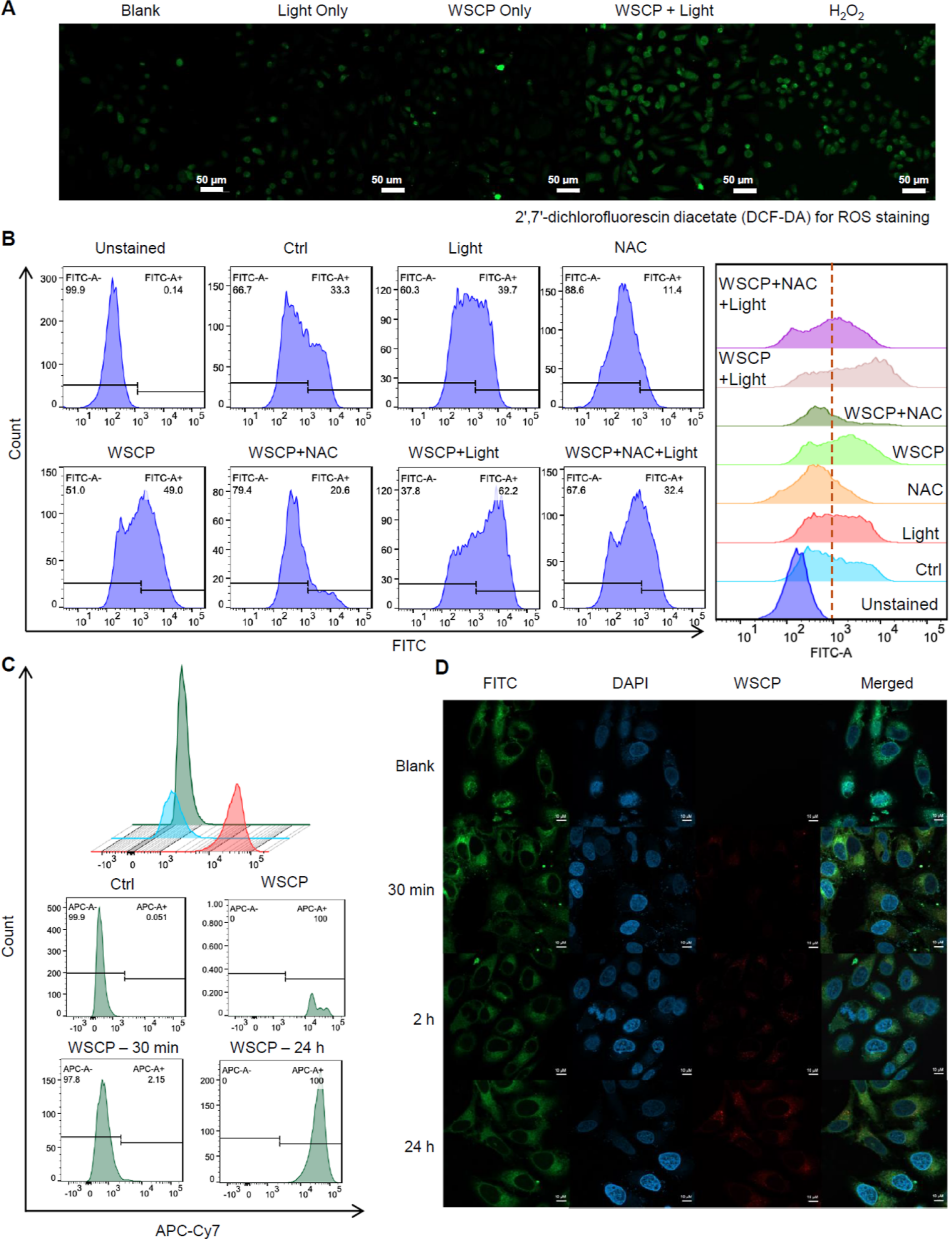
Study of intracellular ROS generation capability and cellular uptake of recombinant WSCP: (A) confocal microscopy images of HeLa cells after different corresponding treatments, and 2′,7′-dichlorofluorescin diacetate (DCF-DA) was used for intracellular ROS staining. Scale bar: 50 μm. Images representative of *n* = 3. (B) Flow cytometric analysis of intracellular ROS generation. (C) Flow cytometric analysis of uptake of recombinant WSCP by HeLa cells. (D) Representative images showing the cellular uptake of recombinant WSCP with the passage of time. Scale bar: 10 μm. Images representative of *n* = 2.

In principle, PDT can be used to treat all types of cancers because singlet oxygen (generated in PDT) indiscriminately kills all cancer cells, which is a major characteristic of PDT advantageous over chemotherapy that relies on the selective and specific binding of proteins and drug molecules. Therefore, we believe that PDT using our recombinant WSCP as the photosensitizer could induce cell death in different cell lines. Six tumor cell lines (HeLa, A549, SKOV3, Hep G2, 4T1, and L02) were selected for the MTT assays (Figures 3G and S8–S14), and their IC_50_ values (approximately 50 μg/mL) were found to be approximately 2.5 times higher than that of HeLa cells (approximately 20 μg/mL). Surprisingly, no significant photocytotoxicity against normal liver cells (L02 cells) was observed when the WSCP concentration was below 50 μg/mL (IC_50_ = 112.1 μg/mL). The unexpected selectivity of cancer cells over normal cells might be due to (1) different cell sensitivity to singlet oxygen and (2) the difference in possible cellular uptake of the recombinant WSCP (Figures 4D and S18). This selectivity could reduce damage to normal tissues and would be advantageous in the clinical application of PDT with WSCP. Moreover, control experiments indicated that the recombinant WSCP (up to 3 mg/mL) was nontoxic against these six cell lines after 48 h in the dark (Figures S9–S15).

### Intracellular Reactive Oxygen Species Generation

Our preliminary results have demonstrated the effectiveness of recombinant WSCP as a photosensitizer in living cells for light-induced cell death. The cell-killing ROS from WSCP/light could be generated in the extracellular fluid, inside the cells (if cellular uptake of WSCP indeed occurred), or both. To investigate the possibility of intracellular ROS generation for cell killing, we used confocal imaging and flow cytometric analysis to examine the photodynamic effect of the recombinant WSCP on the intracellular ROS level in live cells, using 2′,7′-dichlorofluorescin diacetate (DCF-DA) as an ROS-responsive dyes. The intensity of green fluorescent light is proportional to the quantity of in situ generated intracellular ROS. Confocal images (Figures 4A and S16) revealed that the recombinant WSCP and light together were essential and capable of generating significant amounts of intracellular ROS, comparable to the levels observed with hydrogen peroxide (H_2_O_2_) treatment. Flow cytometric analysis provided a more quantitative evaluation of the photoinduced intracellular ROS generation (Figure 4B). We found that the combination of WSCP and red light (660 nm) significantly increased the percentage of HeLa cells with intracellular ROS(62.2%) compared to control conditions (33.3%, 39.7% with light alone, 11.4% with NAC, 49.0% with WSCP alone, 20.6% with WSCP/NAC, and 32.4% with WSCP/NAC/light). This demonstrated the ability of WSCP/light to generate intracellular ROS for cell killing. This finding is significant for understanding the mechanism of action: the combination of WSCP and light likely generates both extracellular and intracellular ROS for cell killing.

### Cellular Uptake Analysis

To further support the generation of ROS inside cells by WSCP/light, we performed cellular uptake experiments with WSCP. The fluorescence emission property of recombinant WSCP enabled a straightforward in vitro study of its cellular uptake by using flow cytometry (Figure 4C). After incubation with WSCP for 30 min, 2.15% of HeLa cells contained WSCP, indicating the cellular uptake through endocytosis. After 24 h of incubation, all HeLa cells (100%) contained WSCP, further supporting time-dependent endocytosis. Confocal microscopy images (Figure 4D) suggested a time-dependent cellular uptake process of WSCP by tumor cells (HeLa) and normal liver cells (L02) (Figure S18). With increasing the coincubation time, more WSCP (red fluorescence) was observed in the cytoplasm (green fluorescence) of HeLa cells: only weak intracellular red fluorescence was detected after 30 min of incubation, and the intensity of red fluorescence increased significantly after 2 and 24 h. It was noteworthy that cellular uptake of WSCP was lower in L02 than in HeLa cells after 24 h of coincubation, which could partially explain the selectivity of WSCP toward cancer cells over normal cells (Figure 3G). The higher uptake of WSCP in tumor cells (HeLa) compared to normal cells (L02) (Figures 4D and S18) might be attributed to several factors related to the unique characteristics of cancer cells and WSCP. First, tumor cells generally exhibit enhanced cell proliferation compared to normal cells, leading to increased nutrient demand and greater endocytic activity.^57−59^ This heightened endocytosis facilitates the uptake of larger biomolecules such as the WSCP. The relatively large size of these complexes is likely better accommodated within the endocytic pathways of proliferating tumor cells. Furthermore, the stability of WSCP under harsh conditions, including the acidic environment of endosomes or lysosomes, ensures that it remains intact and functional, promoting efficient uptake and retention in tumor cells. This contrasts with those of other molecules that may degrade under similar conditions. Additionally, tumor cell membranes often exhibit increased permeability, which can be attributed to alterations in lipid composition and the upregulation of specific transporters.^60,61^ These modifications enhance the ability of tumor cells to take up large, hydrophilic molecules, such as WSCP, which would otherwise have limited access to normal cells with less permeable membranes. We also found that the cell membrane and nuclei of the unfixed HeLa cells with intracellular WSCP were significantly damaged upon 647 nm light excitation (Figure S19), consistent with the in situ generation of extracellular and intracellular ROS for cell killing.

### In Vivo Anticancer Activity

**Figure 5:**
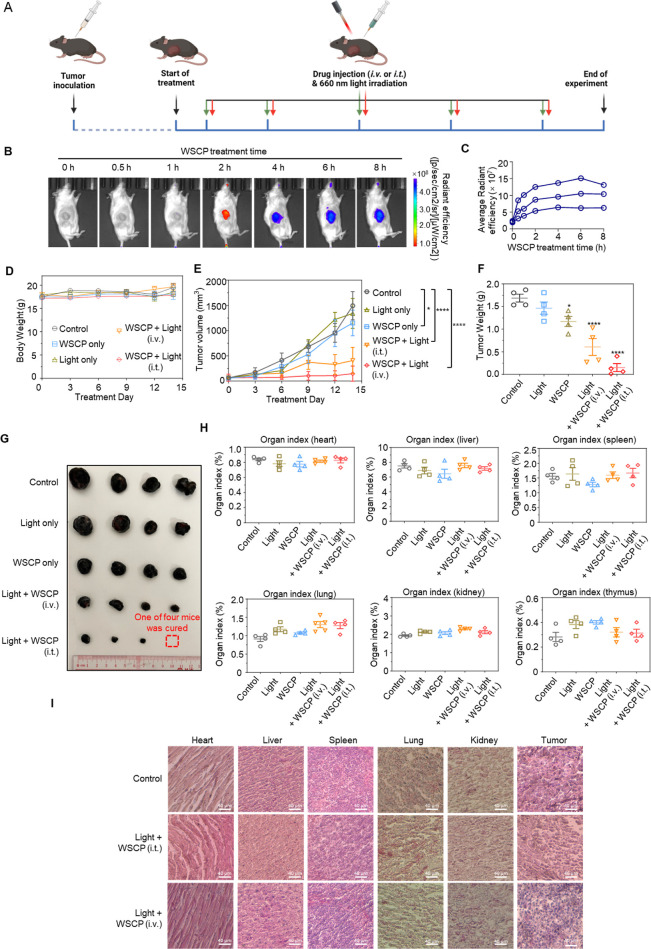
In vivo evaluation of recombinant WSCP in the tumor xenograft mouse model: (A) schematic illustration of in vivo photoinduced anticancer activity evaluation of recombinant WSCP. (B) Real time fluorescence images of mice after the injection of WSCP and the corresponding average radiant efficiency in tumor sites. (C) Statistics of B (*n* = 3). (D) Change of body weight of mice during the treatment. Data were expressed as the mean ± SD (*n* = 4). (E) Tumor volume change of mice during the treatment. Data were expressed as the mean ± SD (*n* = 4). (F) Tumor weight of the killed mice after treatment. (G) Photo of tumor of the killed mice after treatment. (H) Organ index of heart, liver, spleen, lung, kidney, and thymus tissue of different treatment groups. (I)H&E-stained histological section of the organs in each treatment group, *n* = 4 per group. The results were expressed as the mean ± SEM (*p < 0.05, **p < 0.01, ***p < 0.001, ****p < 0.001).

Encouraged by the excellent photoinduced cytotoxicity of the recombinant WSCP against cancer cells, we evaluated its in vivo phototherapeutic efficacy in tumor suppression. Mice with B16F10 melanoma xenografts were used as a subcutaneous tumor model (Figure 5), and both intratumoral injection (i.t.) and intravenous injection (i.v.) were employed to assess its phototherapeutic efficacy and systemic toxicity (Figure 5A). As shown in Figure 5B,C, WSCP rapidly accumulated in the tumor parenchyma and remained at a high level in 4 h. Body weight and tumor size were monitored every 3 days for 14 consecutive days (Figure 5D,E). As shown in Figure 5D, the body weight of mice (18 g) in the control group did not vary significantly during the experimental period, indicating negligible systemic toxicity of the recombinant WSCP. For mice treated with saline, light irradiation alone, or WSCP (i.t.) alone, only limited or no tumor growth suppression was observed: the tumor volumes of nearly all mice in these three groups exceeded 1000 mm^3^ by day 14, compared to less than 100 mm^3^ at day 0. In contrast, mice treated with WSCP (i.t. or i.v.) plus 5 min of light irradiation at 4 h postinjection exhibited significantly slower tumor growth and overall trend of growth arrest after three treatments from day 9. Moreover, nearly all tumors in the i.t. Group remained at a small volume (<100 mm^3^) throughout the therapeutic process, and one tumor was even eliminated after 14 days of treatment (Figure 5E). The weights (Figure 5F) and images of tumor tissues (Figure 5G) yielded consistent results: the tumor volumes in the treated groups were smaller than those in the control group. This provides direct evidence of the in vivo efficacy of the recombinant WSCP as a photosensitizer for tumor suppression. We observed a slight difference in therapeutic performance between the i.t. and i.v. Injection methods, with the latter (i.v.) showing lower efficacy, presumably due to in vivo proteolysis and slow intravenous infusion. In addition, when the tumor tissues were stained for Ki67, a marker of proliferation in oncology, the percentage of Ki67 positive cells in WSCP-light-treated tumor tissues was 55.13 ± 1.31%, approximately 40% lower than that of the vehicle condition (91.30 ± 7.01%; Figure S20). To evaluate the general impact of PDT on healthy tissues and organs, we performed a histological analysis. The main organ tissues (heart, liver, spleen, lung, and kidney) of all mice were collected and examined. Comparison of main organ index (Figure 5H) and hematoxylin and eosin (H&E) stained tissue sections (Figure 5I) from the four experimental groups with the control group indicated no obvious drug-induced injury on these tissues, supporting the high biocompatibility and nontoxicity of the recombinant WSCP as a new class of photosensitizers for PDT. These findings would greatly facilitate the clinical application of PDT using WSCP as a stable, efficient, water-soluble photosensitizer for tumor ablation.

## Conclusions

In summary, we successfully produced WSCPs using heterologous bacterial expression and in vitro reconstitution with chlorophylls from spinach extracts. These recombinant WSCPs exhibited the same chemical–physical properties (photo/thermo-stability and photosensitizer) as those obtained from plants. These unique chemical–physical properties are exploited for the first time in photodynamic therapy to overcome the common limitations of many synthetic organic photosensitizers such as water solubility, photo/thermo-stability, and/or biocompatibility. The efficacy of WSCPs as a new class of photosensitizers in living cells for PDT was evaluated extensively in vitro and finally validated in an in vivo mouse model with total tumor elimination. The recombinant WSCP features the complexation of water-soluble proteins and organic photosensitizers (chlorophylls), which leads to the formation of tetrameric protein-chlorophyll complexes that greatly stabilize the unstable chlorophylls with the retention of their ability as photosensitizers to generate ROS (singlet oxygen) upon light irradiation. This represents the first example of protein-based photosensitizers for PDT and might ignite interest in developing novel protein-based photosensitizers for PDTs via chemical editing and protein engineering.

## Materials and Instruments

### Materials

All reagents and chemicals were purchased from Sigma-Aldrich, Thermo Fisher Scientific, Gibco, Energy Chemical, and the Beyotime Institute of Biotechnology, unless otherwise noted.

### Cell Lines

HeLa cells, A549 cells, SKOV3 cells, Hep G2 cells, 4T1 cells, and B16F10 cells were obtained from the American Type Culture Collection (ATCC). L02 cells were a gift from Prof. Ying CHAU’s lab at HKUST. The HeLa cells, SKOV3 cells, Hep G2 cells, L02 cells, and B16F10 cells were maintained in Dulbecco’s modified Eagle’s medium (DMEM; Gibco, Invitrogen) with 10% FBS, 1% penicillin/streptomycin. 4T1 cells and A549 cells were cultured in the Roswell Park Memorial Institute 1640 (Gibco, Invitrogen) medium with 10% FBS (Gibco, Invitrogen) and 1% penicillin/streptomycin (Gibco, Invitrogen). All cells were cultured in a humidified atmosphere containing 5% CO_2_ at 37 °C.

### Bacteria

The *E. coli* strain BL21 (DE3) harboring PET22b/WSCP was used for WSCP expression.

### Animal Studies

All animal studies were conducted in accordance with the guidelines set by The Government of the Hong Kong Special Administrative Region Committee Department of Health, and the overall project protocols were approved by the Animal Ethics Committee, VPRDO, The HKUST. Female C57BL/6J mice (5–6 week-old) were purchased from the Laboratory Animal Facility (CWB) of HKUST. Mice were housed in a temperature-controlled (22 °C) room with 12 h dark–light cycles and 40–70% humidity.

### Instruments

Ultraviolet–visible (UV–vis) absorbance was measured by using a PerkinElmer Lambda 365 spectrophotometer. Fluorescence spectra were recorded by using an Edinburgh Instrument FS5 fluorescence spectrometer. For photothermal performance analysis, samples were irradiated with 660 nm red light (1 W/cm^2^) using PSU-III-LED light (Honkok Technology), and temperature and infrared thermal images were recorded using a FLIR C3 thermal camera (Teledyne Technologies). For in vitro phototherapy, cells and bacteria were irradiated by using cellular photocytotoxicity irradiators (Shenzhen PURI Materials Technologies). Cells and bacteria viabilities were measured using a FlexStation Multimode microplate reader, and the obtained data were proceeded and analyzed by using Graph Pad Prism 9 software. Flow cytometric analysis was performed using a BS Aria III flow cytometer and analyzed using FlowJo software v10. Confocal images were obtained using a Zeiss LSM 980 confocal microscope with AiryScan2 and proceeded with ZEN 2009 software. For in vivo anticancer phototherapy, tumors in mice were irradiated with 660 nm red light (152 mW/cm^2^) by using a Fiber Coupled Laser System (Beijing Blueprint Electronic Technology). Photos of wound healing assay and organ tissue slices were captured by a Nikon Ts2 Eclipse light microscope.

## Experimental Procedure

### Expression, Reconstitution, and Purification of the Recombinant WSCPs

WSCPs were produced in *E. coli* strain BL21 (DE3) harboring PET22b/WSCP. The expression culture in the LB medium containing ampicillin (100 μg/mL) was inoculated at 37 °C and 220 rpm and grown until OD600 reached 0.6–0.8. Protein expression was induced by adding isopropyl β-d-1-thiogalactopyranoside (IPTG) (300 μM) at 37 °C. After 4 h, the cell pellet was harvested by centrifugation. To assemble WSCPs with chlorophyll, an excess amount of spinach extract (prepared according to reported methodology)^38,62^ was added to cell pellet in lysis buffer (300 mM NaCl, 100 mM Tris–HCl, 0.1 mM PMSF, pH 7.5). The mixture was sonicated to lyse the cells and separate the spinach extract. The homogenized lysate was centrifuged (18,000 rpm, 45 min, 4 °C) to collect supernatant as a transparent green solution. The reconstituted WSCP was purified using Ni-NTA chromatography with wash buffer (300 mM NaCl, 100 mM Tris, 50 mM imidazole, pH 7.5, 8 CV) and elution buffer (300 mM NaCl, 100 mM Tris, 500 mM imidazole, pH 7.5, 3 CV). The purified protein was dialyzed against Milli-Q water (4 L × 5), concentrated, and then stored at −80 °C after being flash-freezed in liquid nitrogen. Protein purity was assessed by using SDS-PAGE.

### Singlet Oxygen Generation Efficiency Measurement and Calculation

The singlet oxygen indicator 9, 10-anthracenediyl-bis (methylene)dimalonic acid (ABDA) was employed to measure the singlet oxygen generation of the recombinant WSCP and two commercial photosensitizers, RB and Ce6, upon light irradiation. The absorbance of each sample (*c* = 1 × 10^–4^ M) was first set as the blank. ABDA (*c* = 2 × 10^–4^ M) was then added to each sample in a dark room, and the UV absorbance of the samples was measured immediately. The sample mixture was irradiated with white light (1 mW/cm^2^) or 660 nm red light (7 mW/cm^2^) at 30 s intervals for 2 min. The UV absorption of ABDA at 378 nm was recorded at various time points to determine the decay rate of the photosensitizing process. The singlet oxygen generation efficiencies (ΦROS) of photosensitizers upon light irradiation were measured and calculated using a modified method based on Liu’s report:^54^ The singlet oxygen generation efficiency (ΦROS) was defined as the ABDA decomposition calculated as ln(*A*_0_/*A*), where *A*_0_ and A are the absorbances of ABDA at 378 nm before and after light irradiation.

### Visualization of the Photothermal Conversion Performance of the Recombinant WSCP

To visualize the photothermal conversion performance of the recombinant WSCP, infrared thermal images of recombinant WSCP (2 mg/mL) in water were captured using an infrared thermal camera.

### Photothermal Conversion Performance of Recombinant WSCPs at Different Concentrations in H_2_O upon Irradiation

The photothermal conversion performance of the recombinant WSCP was investigated in a 1.5 mL tube containing 200 μL of the recombinant WSCP at different concentrations (1, 2, and 3 mg/mL) in H_2_O under 660 nm red light (0.8 W/cm^2^) irradiation for 10 min. The temperatures of each sample was recorded every minute.

### Photothermal Stability of the Recombinant WSCP

The photothermal stability of the recombinant WSCP (3 mg/mL) was evaluated using 660 nm red light (0.8 W/cm^2^) irradiation for four on/off cycles, with each cycle lasting 20 min (10 min light on and 10 min light off). The temperatures of each sample was recorded every minute.

### Long-Term Stability Evaluations of Free Chlorophylls and the Recombinant WSCP at Ambient

The recombinant WSCP (2 mg/mL in H_2_O), free chlorophylls (2 mg/mL) in H_2_O, and free chlorophylls (2 mg/mL) in MeOH were stored in 1.5 mL tubes and exposed to normal room light at ambient temperature for 28 days. The status of the samples was recorded daily by using by photography.

### In Vitro Anticancer Phototherapy (Cell Viability)

For in vitro cytotoxicity evaluation, cells were cultured in a 96-well plate at a density of 0.8 × 10^4^ cells/well at 37 °C in a humidified atmosphere with 5% CO_2_ for 24 h. Subsequently, the cultured medium was removed, and the cells were incubated with fresh medium containing the recombinant WSCP at different concentrations for another 24 h. The cells were then exposed to 660 nm light (63.3 mW/cm^2^) for 30 min. For nonirradiated groups, the cells were kept in the dark for 30 min. After different treatments, the cells were incubated at 37 °Cin a humidified atmosphere with 5% CO_2_for 24 h. Subsequently, 10 μL of 3-(4,5-dimethylthiazol-2-yl)-2,5diphenyltetrazolium bromide (MTT) (5 mg/mL) was added to each well, and the cells were incubated at 37 °C in a humidified atmosphere with 5% CO_2_ for 4 h. After the medium was removed, 100 μL of DMSO was added to each well to dissolve the precipitated formazan. Finally, the absorption of each well at 490 nm was measured by a FlexStation Multimodel microplate reader. Data were processed and analyzed by using Graph Pad Prism 9 software.

### Colony Formation

To evaluate the photoinduced antiproliferation effect of WSCPs, a colony formation assay was performed. Cells were seeded in 6-well plates at a density of 1 × 10^3^ cells/well at 37 °C in a humidified atmosphere with 5% CO_2_ for 24 h. Subsequently, the cultured medium was removed, and the cells were incubated with fresh medium containing the recombinant WSCP with different concentrations for another 24 h; then, the cells were exposed with 660 nm light (31.8 mW/cm^2^, 15 min). For nonirradiated groups, the cells were stored in the dark. After treatment, the cells were incubated for another overnight and then washed with PBS. The cells were cultured for 14 days to form colonies, and the medium was changed every 3 days. After colony formation, the colonies were washed with PBS, fixed with 4% paraformaldehyde (PFA) for 10 min, and then stained with 0.2% crystal violet solution in 10% ethanol for 20 min before the photo being taken by using a Nikon Eclipse Ts2 light microscope.

### Wound Healing Assay

For wound healing assay, the cells were seeded in 6-well plates at a density of 1 × 10^6^ cells/well at 37 °C in a humidified atmosphere with 5% CO_2_ for 24 h. After creating a straight scratch with a sterile plastic tip, the cells were incubated with fresh medium with or without the recombinant WSCP (25 μg/mL) for another 24 h. Subsequently, the cells were exposed to 660 nm light (31.8 mW/cm^2^, 15 min). For nonirradiated groups, the cells were kept in the dark for 15 min. After treatments, the cells were incubated for 24 h and then washed with PBS. Finally, the cells were fixed with 4% PFA for 10 min and then stained with 0.2% crystal violet solution in 10% ethanol for 20 min before the photo being taken. Images were obtained using a Nikon Eclipse Ts2 light microscope.

### Verification of the ROS Production Effect of the Recombinant WSCP

HeLa cells were cultured in a 96-well plate at a density of 0.8 × 10^4^ cells/well at 37 °Cin a humidified atmosphere with 5% CO_2_ for 24 h. Subsequently, the cultured medium was removed, and the cells were incubated with 100 μL of fresh medium containing *N*-acetyl-l-cysteine (NAC) (5 mM) the ROS scavenger for 2 h, and then, the medium was removed and the cells were added to the fresh medium containing both NAC (5 mM) and the recombinant WSCP with different concentrations and incubated for another 24 h. Subsequently, the cells were exposed with 660 nm light (63.3 mW/cm^2^) for 30 min. For nonirradiated groups, the cells were kept in the dark for 30 min. After different treatments, the cells were incubated at 37 °C in a humidified atmosphere with 5% CO_2_ for 24 h. Subsequently, 10 μL of MTT (5 mg/mL) was added to each well, and the cells were incubated at 37 °C in a humidified atmosphere with 5% CO_2_ for 4 h. After removal of medium, 100 μL of DMSO was added to each well to dissolve the precipitated formazan. Finally, the absorption of each well at 490 nm was measured.

### Evaluation of Dark Toxicity of the Recombinant WSCP on HeLa Cells

HeLa cells was used as a model for evaluation of the dark toxicity of the recombinant WSCP. The cells were cultured in a 96-well plate at a density of 0.8 × 10^4^ cells/well at 37 °C in a humidified atmosphere with 5% CO_2_ for 24 h. Subsequently, the cultured medium was removed, and the cells were incubated with fresh medium containing the recombinant WSCP with different concentrations for another 48 h. After treatment, 10 μL of MTT (5 mg/mL) was added to each well, and the cells were incubated at 37 °C in a humidified atmosphere with 5% CO_2_ for 4 h. After removal of the medium, 100 μL of DMSO was added to each well to dissolve the precipitated formazan. Finally, the absorption of each well at 490 nm was measured. Data were expressed as the mean ± SD with three independent experiments.

### Intracellular ROS Measurement

To evaluate cellular ROS generation of the recombinant WSCP, we utilized inverted confocal microscopy and flow cytometric analysis with 2′,7′-dichlorofluorescin diacetate (DCF-DA) as an indicator to probe the intracellular reactive oxidative species generated. The DCF-DA was excited at 488 nm, and the emission was collected at 500–530 nm. No background fluorescence of cells was detected under the setting condition. For flow cytometry, HeLa cells were seeded in 6-well plates at a density of 1 × 10^6^ cells/well. After treatments, cells were incubated with DCF-DA (5 μM) for 15 min before cell collection and analyzed by BD Aria III flow cytometer through FlowJo software v10. From each sample, 1 × 10^4^ cells were counted. For confocal microscopy, cells were seeded in confocal dishes at a density of 1 × 10^6^ cells/dish and incubated with WSCP (150 μg/mL) for different time. Subsequently, after light irradiation, the cells were incubated with DCF-DA (5 μM) for 15 min. Then, the cells were washed with fresh medium before confocal analysis. Confocal images were obtained by using a Zeiss LSM 980 confocal microscope with AiryScan2 and analyzed by ZEN 2009 software.

### Cellular Uptake Analysis by Flow Cytometry

For flow cytometry, HeLa cells were seeded in 6-well plates at a density of 1 × 10^6^ cells/well and incubated with WSCPs for different incubated time. The cells were washed with PBS and then collected in tubes, and intracellular WSCP (150 μg/mL) was detected by using APC-Cy7 channel through BD Aria III flow cytometer. From each sample, 1 × 10^4^ cells were counted.

### Observation of the Recombinant WSCP through a Confocal Microscope

For verification of the hypothesis of using a confocal microscope to detect the intracellular WSCP, the HeLa cells were used as models for preliminary attempts. The HeLa cells were seeded in confocal dishes at a density of 1 × 10^6^ cells/dish and incubated at 37 °C in a humidified atmosphere with 5% CO_2_ for 24 h. After removal of the medium, cells were added to free medium with WSCP (150 μg/mL) and observed through a confocal microscope immediately. The recombinant WSCP was excited at 647 nm, and the emission was collected at 680–800 nm. With the time going upon the irradiation during the observation, cell morphology would change, and cells would be killed upon the irradiation by confocal instrument.

### Cellular Uptake Analysis by Confocal Microscope Analysis

For confocal microscopy analysis of the recombinant WSCP cellular uptake, cells were seeded in dishes (containing a coverslip for cell adhesion) at a density of 1 × 10^6^ cells/dish, and then, the cells incubated with WSCPs (150 μg/mL) for indicated incubated time. After washing with PBS, the cells were loaded with CellMask Green Plasma Membrane Stain for 15 min at room temperature to stain the cell membrane followed by washing with PBS and fixing by using 4% paraformaldehyde for 10 min. Finally, after washing with PBS twice, the coverslip was mounted with ProLong Gold Anti-Fade containing 4′,6-diamidino-2-phenylindole (DAPI) to stain the nucleus before confocal analysis. Confocal images of cells were obtained by using a Zeiss LSM 980 confocal microscope with AiryScan2 and analyzed by ZEN 2009 software. The recombinant WSCP was excited at 647 nm, and the emission was collected at 680–800 nm. CellMask Green Plasma Membrane Stain was excited at 488 nm, and emission was collected at 500–570 nm. DAPI was excited at 356 nm, and emission was collected at 400–500 nm.

### Observation of Unfixed HeLa Cells with Intracellular Recombinant WSCPs through a Confocal Microscope

HeLa cells were seeded in dishes (containing a coverslip for cell adhesion) at a density of 1 × 10^6^ cells/dish and incubated with or without WSCPs (150 μg/mL) for different incubated time. After washing with PBS, the cells were stained with or without CellMask Green Plasma Membrane Stain dye for 15 min at room temperature to stain the cell membrane. After washing with PBS, the coverslip was mounted with ProLong Gold Anti-Fade containing 4′,6-diamidino-2-phenylindole (DAPI) to stain the nucleus before confocal analysis without any cell fixation. For the group, that staining membrane alone was used directly after washing with PBS without mounting with DAPI. Confocal images of cells were obtained by using a Zeiss LSM 980 confocal microscope with AiryScan2 and analyzed by ZEN 2009 software. The recombinant WSCP was excited at 647 nm, and the emission was collected at 680–800 nm. CellMask Green Plasma Membrane Stain was excited at 488 nm, and emission was collected at 500–570 nm. DAPI was excited at 356 nm, and emission was collected at 400–500 nm.

### In Vivo Fluorescence Imaging of WSCP Biodistribution

Tumor-bearing mice (*n* = 3) were under general anesthesia (2.5% isoflurane/O_2_) and on the right side of the mice and positioned in the dorsal recumbency. Baseline fluorescence signals were acquired at 0 h (preinjection control) using the IVIS spectrum in vivo imaging system (PerkinElmer) with the following parameters: 675 nm excitation/740 nm emission filters and 10 s exposure time. Following baseline imaging, mice received intravenous administration of WSCP (30 mg/kg) through the tail vein. Serial fluorescence imaging was subsequently performed at 0.5, 1, 2, 4, 6, and 8 h postinjection using identical imaging settings. Quantitative analysis was conducted by defining consistent regions of interest over tumor sites using Living Image 4.4 software, with average fluorescence intensity calculated for each time point.

### In Vivo Anticancer Phototherapy

All animal experiments were performed in compliance with institutional animal care guidelines and according to committee-approved protocols. For construction of tumor-bearing mice, 100 μL of free FBS medium with 1 × 10^6^ B16F10 cells was injected into the right flank of 5–6 weeks old C57BL/6J female mice. The size of the transplanted tumor xenograft was measured until its long diameter was around 60 mm^3^. Mice were randomly assigned to five groups (four mice per group): vehicle (treated with 100 μL PBS via intratumoral injection), light irradiation alone (treated with 100 μL PBS via intratumoral injection and 152 mW/cm^2^ 660 nm light irradiation for 5 min at 4 h post injection), WSCP alone (treated with 30 mg/kg WSCP via intratumoral injection), and two experiment groups (treated with 30 mg/kg WSCP via intratumoral injection or intravenous injection respectively, and 152 mW/cm^2^ 660 nm light irradiation for 5 min at 4 h post injection). All treatments were performed on days 1, 3, 6, 9, and 12. The body weight and tumor volume were recorded every 3 days for 14 days. The tumor volume was calculated by using the following formula: tumor volume (mm^3^) = π/6 × (length) × (width)^2^. After treatment for 14 days, all mice were euthanized and weighted. Tumors and major organs of mice (heart, liver, spleen, lung, kidney, and thymus) were collected and weighed.

### Hematoxylin and Eosin H&E Staining

For histological analysis, major organs (including heart, liver, spleen, lung, kidney) and tumor tissues were harvested from killed mice on the 15th day post-treatment of different treatment conditions and fixed in 4% PFA. The samples were then dried and embedded in paraffin. Before H&E staining, tissue sections were dewaxed in xylene, rehydrated by gradient ethanol, washed with distilled water, and then stained with hematoxylin and eosin (H&E). After staining, the slices were dehydrated with increasing concentrations of ethanol and xylene. The morphological images of the tissues were captured using a light microscope.

### Immunofluorescence (IF) Staining

4% PFA-fixed and paraffin-embedded mice tumors were cut into 4 μm sections. Antigen retrieval was performed by citrate buffer (pH 6.0) for 20 min after deparaffinization and rehydration. Next, the tumor tissue sections were blocked with 5% serum for secondary antibodies for 30 min at room temperature and then incubated overnight at 4 °C with primary antibodies against Ki67 (GB111499, Service). Tissue sections were treated with the appropriate fluorescence-labeled secondary antibodies. DAPI was used for nuclei staining. Images of stained specimens were captured using a Pannoramic MIDI or LSM 980 instrument (ZEISS).

### Purity Determination of WSCPs

The purity of WSCPs was determined by Agilent Infinity 1260 II analytical high-performance liquid chromatography (AdvanceBio SEC 300A (300 × 4.6 mm2, particle size 2.7 μm); 50 mM eluting system; flow rate = 0.35 mL/min with DAD at 280 or 665 nm). The purity of the WSCP is over 95%.

## Supporting Information Available

Amino acid sequence of WSCPs, singlet oxygen generation efficiency, visualization of the photothermal conversion performance, photothermal conversion efficiency calculation, long-term stability evaluations, *in vitro* anticancer phototherapy, colony formation assay, wound healing assay, evaluation of dark toxicity, intracellular ROS measurement, observation of the recombinant WSCP, cellular uptake analysis, observation of unfixed HeLa cells, *in vivo* validation of tumor proliferation, and HPLC data of WSCPs (PDF)
