# Supplementary material for: Cancer Photodynamic Therapy Enabled by Water-Soluble Chlorophyll Protein
Source: ACS Appl Mater Interfaces. 2025 Mar 6;17(11):16668–80. doi: 10.1021/acsami.5c01280 (PMC11931482; doi:10.1021/acsami.5c01280)
Supplement: Supplementary file 1 — am5c01280_si_001.pdf [file am5c01280_si_001.pdf]

Supporting Information

Cancer Photodynamic Therapy Enabled by Water-Soluble Chlorophyll Protein

Lixin Liang[a,b#], Wenjun Wang[b#], Manjia Li[c], Yingjie Xu[c], Zhangdi Lu[f], Jingjing Wei[e], Ben Zhong Tang[b,d], Fei Sun[c]* & Rongbiao Tong[b]*

[a] Guangxi Key Laboratory of Special Biomedicine; School of Medicine, Guangxi University, Nanning, 530004, China.
[b] Department of Chemistry, The Hong Kong University of Science and Technology Clear Water Bay, Kowloon, Hong Kong, China.
[c] Department of Chemical and Biological Engineering, The Hong Kong University of Science and Technology, Clear Water Bay, Kowloon, Hong Kong, China.
[d] School of Science and Engineering, Shenzhen Institute of Aggregate Science and Technology, The Chinese University of Hong Kong, Shenzhen City, Guangdong, 518172, China.
[e] College of Chemical and Environmental Engineering, Anyang Institute of Technology, Anyang 455000, China.
[f] Exponent Ltd., 12 Science Park West Avenue, Unit 802-803, Sha Tin, New Territories, Hong Kong

E-mail (RT): rtong@ust.hk, or E-mail (FS): kefsun@ust.hk
[#]These authors contributed equally to this work.

Table of Contents

1. Supplementary Figures 3

1.1 Amino acid sequence of WSCP 3

1.2 Singlet oxygen generation efficiency measurement and calculation 4

1.3 Visualization of the photothermal conversion performance of the recombinant WSCP 5

1.4 Photothermal conversion efficiency calculation 6

1.5 Long-term stability evaluations of free chlorophylls (Chls) and the recombinant WSCP at ambient. 7

1.6 *In vitro* anticancer phototherapy toward HeLa cells 8

1.7 Colony formation assay 9

1.8 Wound healing assay 10

1.9 *In vitro* anticancer phototherapy toward different cell lines (cell viability) 11

1.10 Evaluation of dark toxicity of the recombinant WSCP on HeLa cells 15

1.11 Intracellular ROS measurement 16

1.12 Observation of the recombinant WSCP through confocal microscope. 17

1.13 Cellular uptake analysis by confocal microscope analysis. 18

1.14 Observation of unfixed HeLa cells with intracellular the recombinant WSCP through confocal microscope. 19

1.15 *In Vivo* Validation of Tumor Proliferation via Ki67 Immunofluorescent Staining in Tumor Sections 20

1.16 HPLC data of WSCP (Purity determination) 21

2. References 22

1. Supplementary Figures

1.1 Amino acid sequence of WSCP

INDEEPVKDTNGNPLKIETRYFIQPASDNNGGGLVPANVDLSHLCPLGIVRTSLPYQPG
LPVTISTPSSSEGNDVLTNTNIATFDAPIWPCPSSKTWTVDSSSEEKYIITGGDPKSGES
FFRIEKYGNGKNTYKLVRYDNGEGKSVGSTKSLWGPALVLNDDDDSDENAFPIKFRE
VDT

1.2 Singlet oxygen generation efficiency measurement and calculation

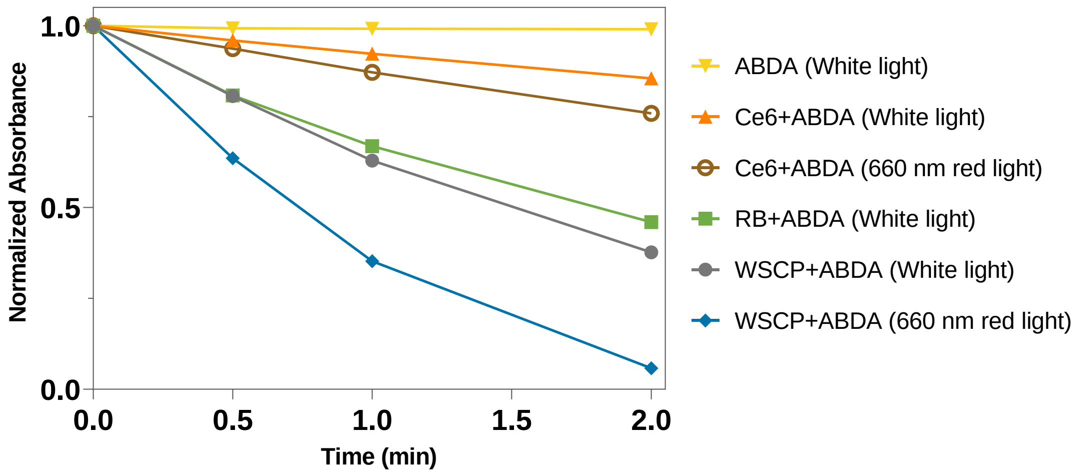

Supplementary Figure S1. Normalized absorptions of 9,10-anthracenediyl-bis(methylene) dimalonic acid (ABDA) ($c = 2\times10^{-5}$ M) in H_2O upon white-light (1 mW/cm^2) or 660 nm red light (7 mW/cm^2) irradiation in the presence of different photosensitizers ($c = 1\times10^{-4}$ M): WSCP, Rose Bengal or Ce6 (porphyrin derivative).

Supplementary Table S1. Singlet oxygen generation efficiencies of different photosensitizers upon light irradiation

Photosensitizer (Light type)	None (White)	RB (White)	Ce6 (White)	Ce6 (Red)	WSCP (White)	WSCP (Red)
Φ_{ROS} [$\times10^{-3}$ s^{-1}]	0.07	6.44	1.30	2.31	8.19	24.1

1.3 Visualization of the photothermal conversion performance of the recombinant WSCP

For visualization of the photothermal conversion performance of the recombinant WSCP, infrared thermal images of recombinant WSCP (2 mg/mL) in H_2O were captured by infrared thermal images camera.

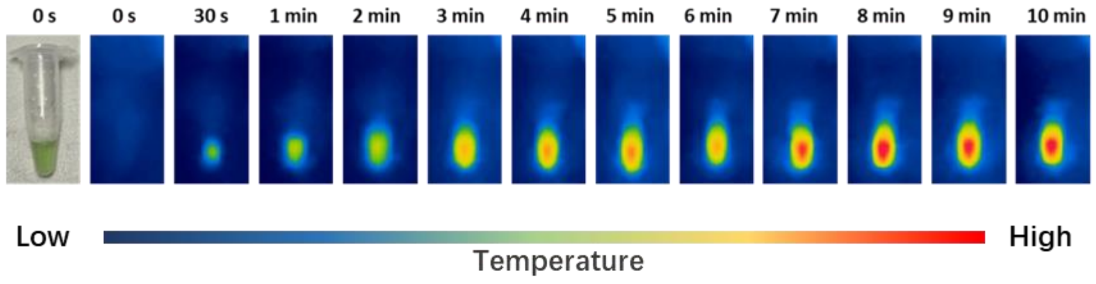

Supplementary Figure S2. Infrared thermal images of recombinant WSCP (2 mg/mL) in H2O upon light irradiations (660 nm, 0.8 W/cm^2). Images representative of n = 3.

1.4 Photothermal conversion efficiency calculation

The photothermal conversion efficiency of the recombinant WSCP was measured according to the reported method[1, 2]:

$$\eta = \frac{hs(T_{max} - T_{surr}) - Q_{Dis}}{I(1 - 10^{-A_{660}})}$$

h is the heat transfer coefficient; *s* is the surface area of the container. Q_{Dis} represents heat dissipated from the laser mediated by the solvent and container. *I* is the laser power and *A* is the absorbance at 660 nm.

$$hs = \frac{mC_{water}}{\tau_s}$$

m is the mass of the solution containing the photoactive material, *C* is the specific heat capacity of the solution (C_{water} = 4.2 J/(g·°C), and τs is the associated time constant.

$$t = -\tau_s ln(\theta)$$

θ is a dimensionless parameter, known as the driving force temperature.

$$\theta = \frac{T - T_{surr}}{T_{max} - T_{surr}}$$

T_{max} and T_{surr} are the maximum steady state temperature (46.8 °C) and the environmental temperature (25.8 °C), respectively.

According to the above reported method, the photothermal conversion efficiency of the recombinant WSCP (3 mg/mL) upon 660 nm red light (0.8W/cm^2) is 16.4%.

1.5 Long-term stability evaluations of free chlorophylls (Chls) and the recombinant WSCP at ambient.

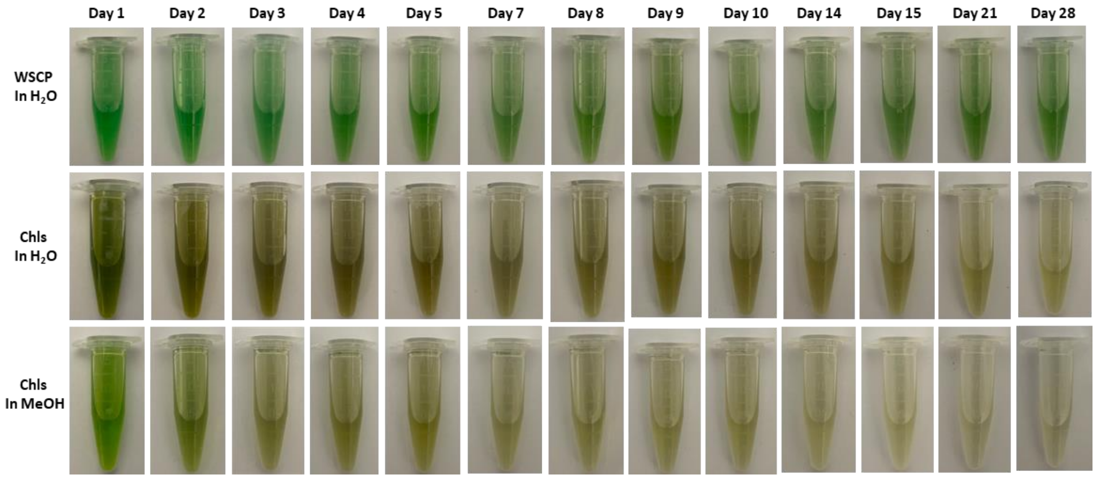

Supplementary Figure S3. Comparison between long-term stabilities of free chlorophylls and recombinant WSCP at ambient condition. Images representative of *n* = 3.

1.6 *In vitro* anticancer phototherapy toward HeLa cells

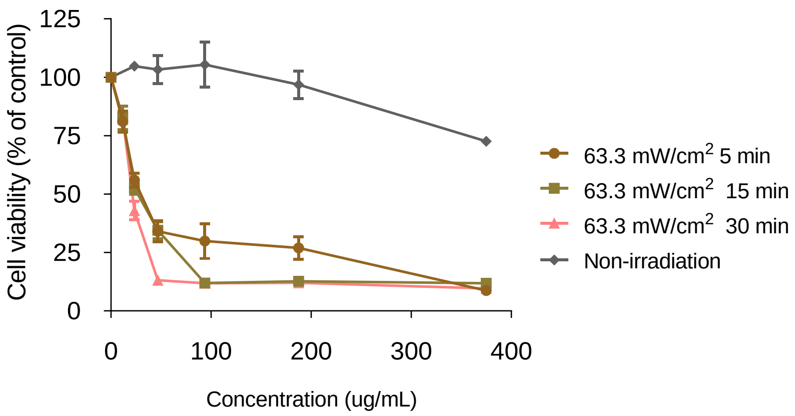

Supplementary Figure S4. Relative viability and IC_{50} of HeLa cells after incubation with recombinant WSCP with or without 660 nm red light irradiation (63.3 mW/cm^2) for different irradiation time. Data were expressed as the mean ± SD (*n* > 3).

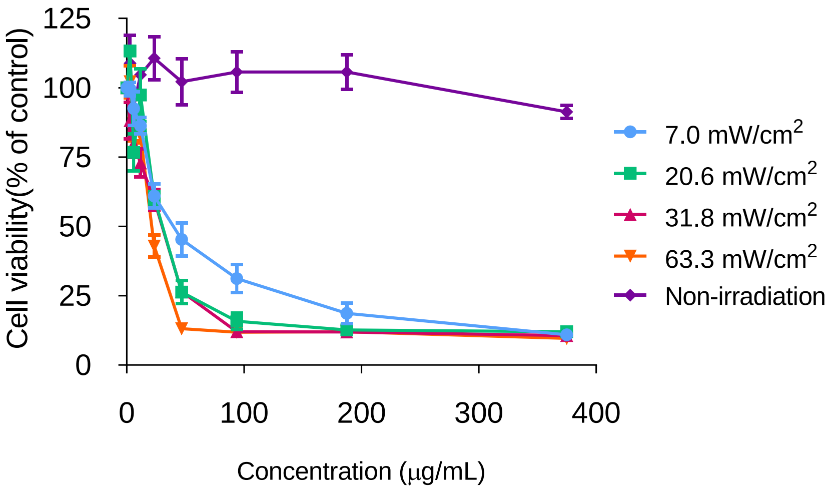

Supplementary Figure S5. Relative viability and IC_{50} of HeLa cells after incubation with recombinant WSCP with or without 660 nm red light irradiation (30 min) with different lighting power density. Data were expressed as the mean ± SD (*n* > 3).

1.7 Colony formation assay

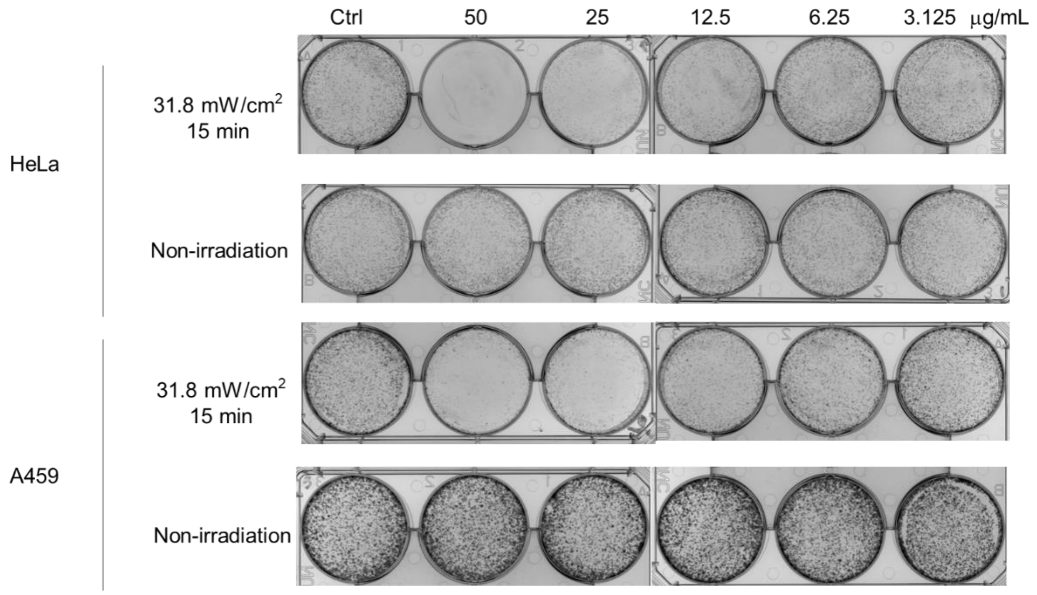

Supplementary Figure S6. Colony formation assay was performed to study the long-term effects of recombinant WSCP photo-induced therapy on the survival and proliferation of HeLa cells and A549 cells. Images representative of *n* = 3.

1.8 Wound healing assay

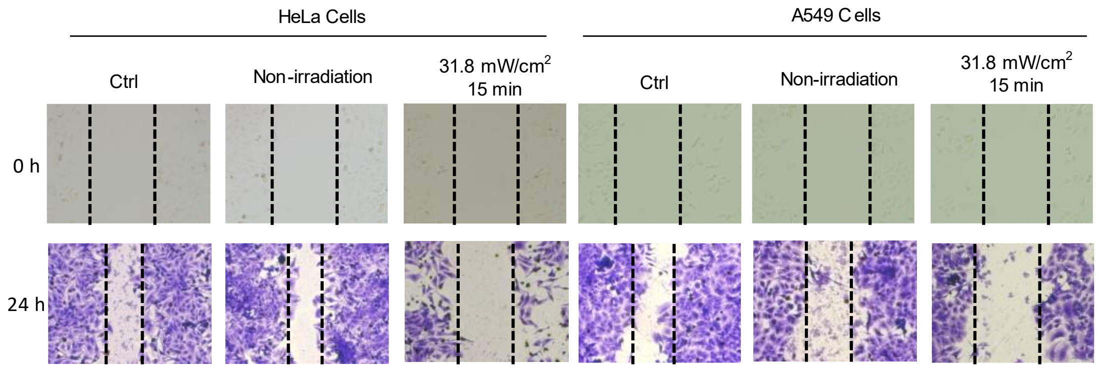

Supplementary Figure S7. Wound healing assay results of HeLa cells and A549 cells. Images representative of *n* = 3.

1.9 *In vitro* anticancer phototherapy toward different cell lines (cell viability)

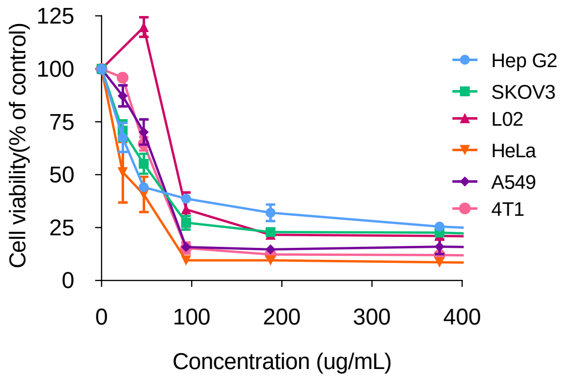

Supplementary Figure S8. In vitro photocytotoxicity of recombinant WSCP toward different cell lines. Data were expressed as the mean ± SD (*n* = 3).

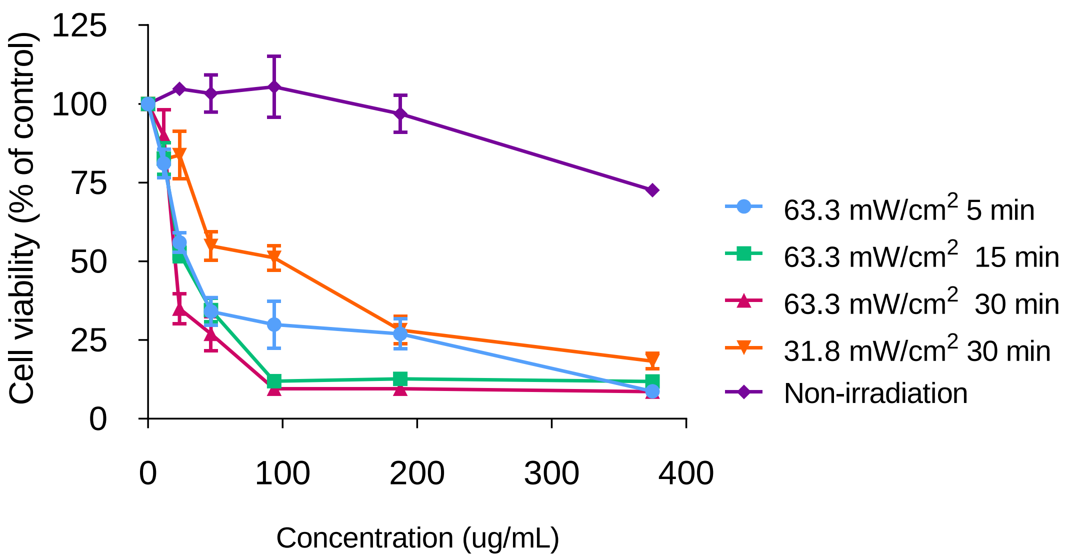

Supplementary Figure S9. Cell viabilities of HeLa cells incubated with the recombinant WSCP upon light irradiation. Data were expressed as the mean ± SD (*n* > 3).

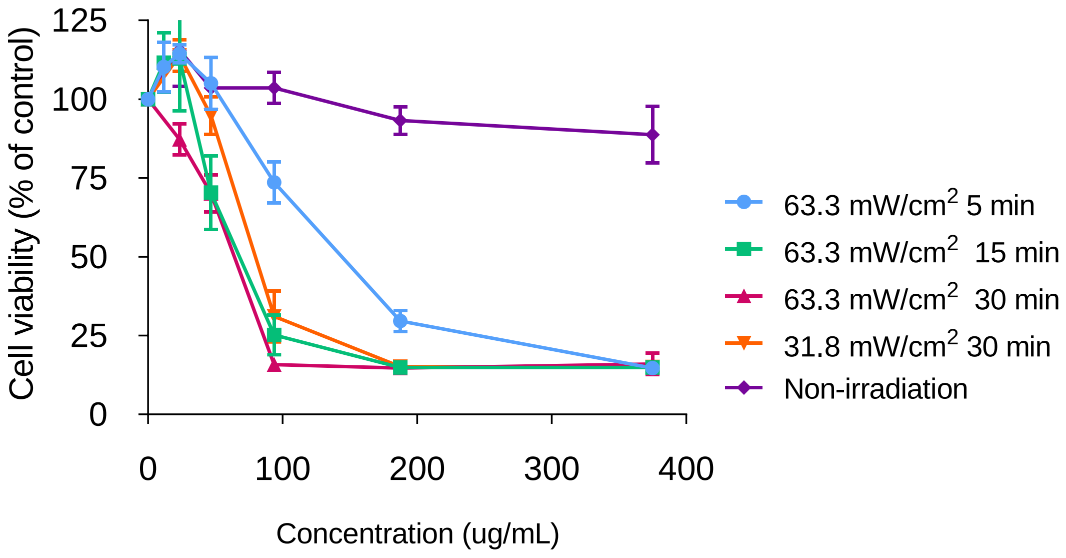

Supplementary Figure S10. Cell viabilities of A549 cells incubated with the recombinant WSCP upon light irradiation. Data were expressed as the mean ± SD (n > 3).

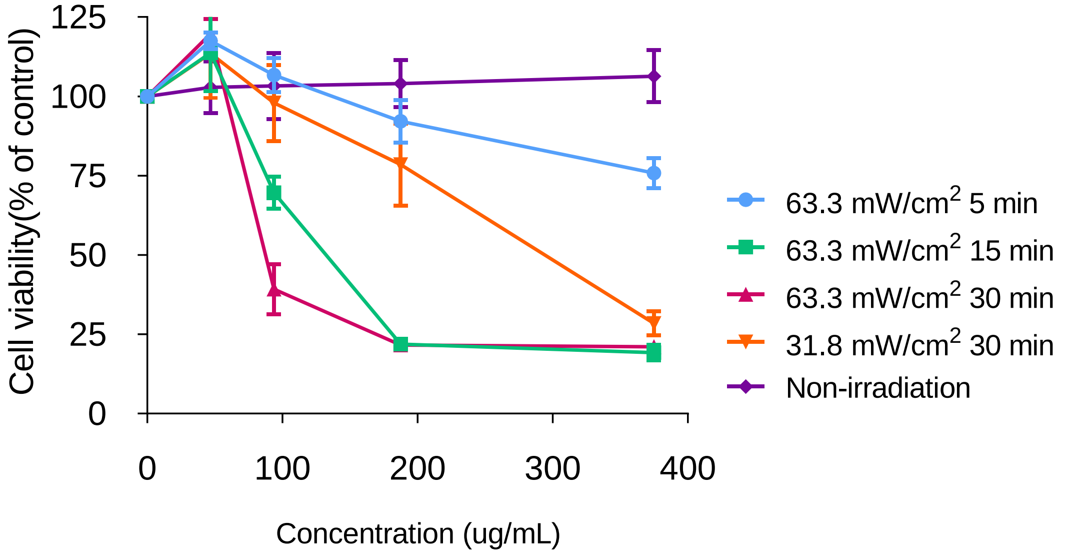

Supplementary Figure S11. Cell viabilities of L02 cells incubated with the recombinant WSCP upon light irradiation. Data were expressed as the mean ± SD (n > 3).

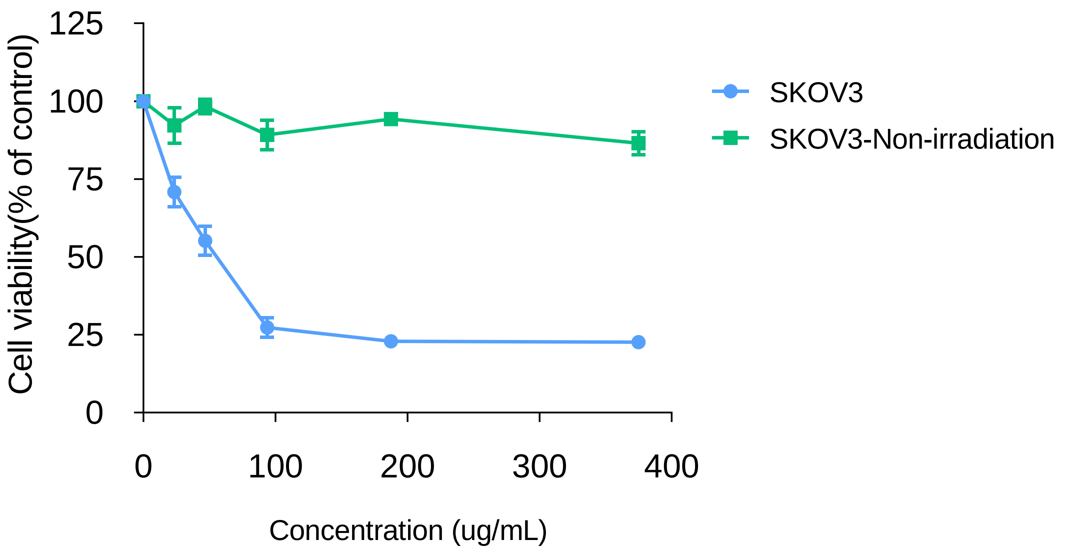

Supplementary Figure S12. Cell viabilities of SKOV3 cells incubated with the recombinant WSCP upon light irradiation. Data were expressed as the mean ± SD (n = 3).

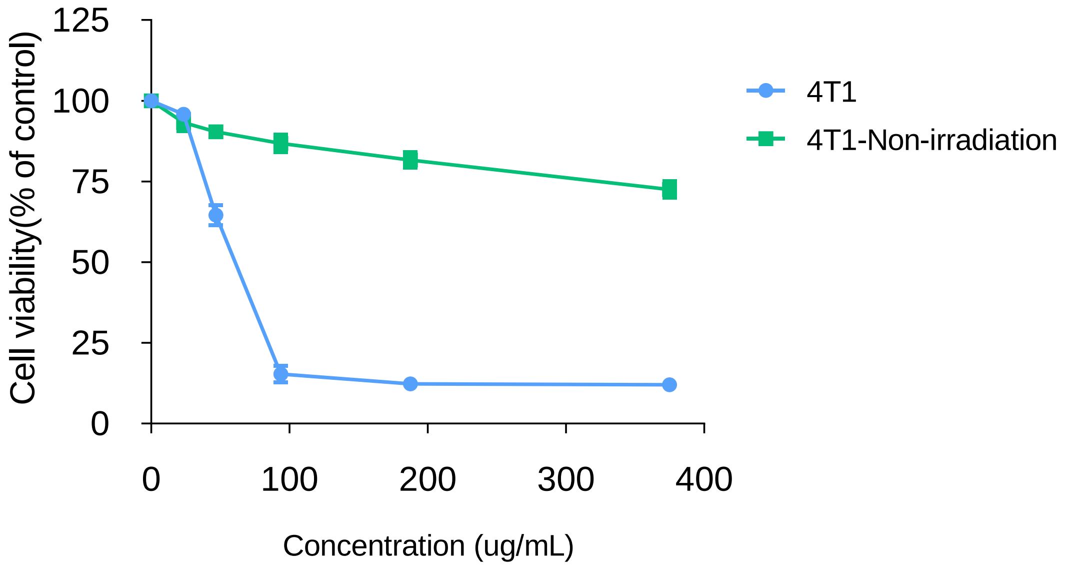

Supplementary Figure S13. Cell viabilities of 4T1 cells incubated with the recombinant WSCP upon light irradiation. Data were expressed as the mean ± SD (n = 3).

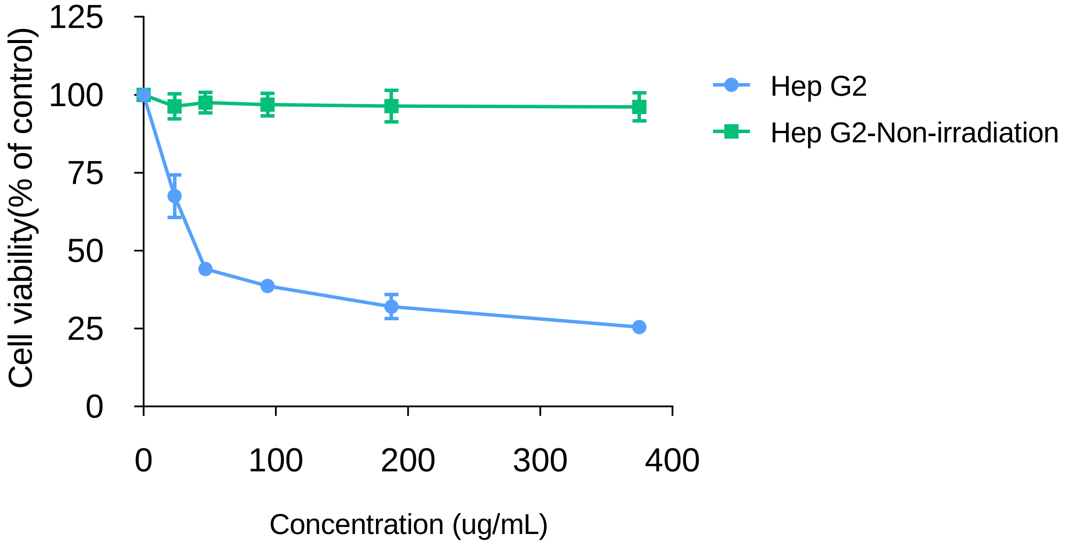

Supplementary Figure S14. Cell viabilities of Hep G2 cells incubated with the recombinant WSCP upon light irradiation. Data were expressed as the mean ± SD (*n* = 3).

1.10 Evaluation of dark toxicity of the recombinant WSCP on HeLa cells

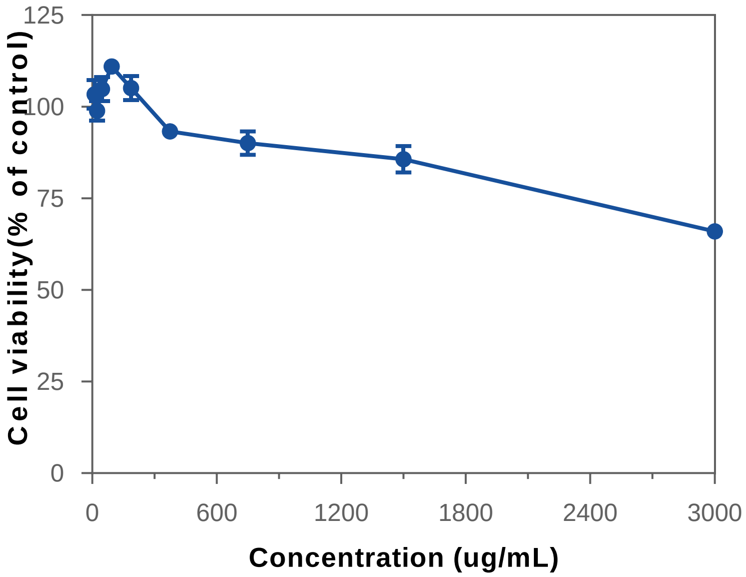

Supplementary Figure S15. Cell viabilities of HeLa cells after incubation with the high concentration WSCP for 48 h in darkness. Data were expressed as the mean ± SD (*n* = 3).

1.11 Intracellular ROS measurement

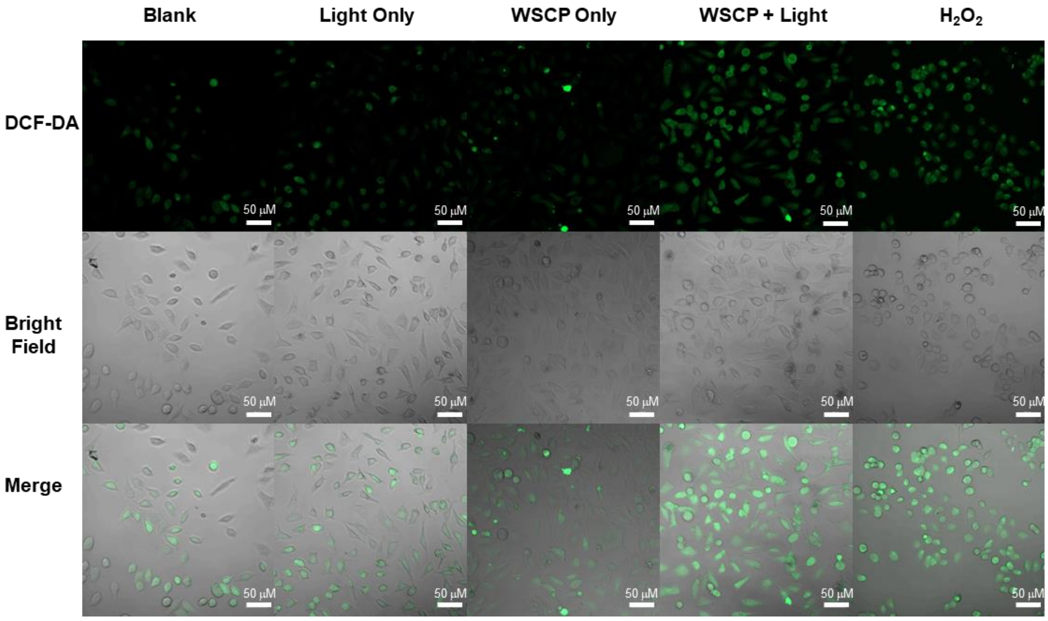

Supplementary Figure S16. Confocal microscopy images of HeLa cells after different corresponding treatments, and 2',7'-dichlorofluorescin diacetate (DCF-DA) was used for intracellular ROS staining. Scale bar: 50 μM. Images representative of n = 3.

1.12 Observation of the recombinant WSCP through confocal microscope.

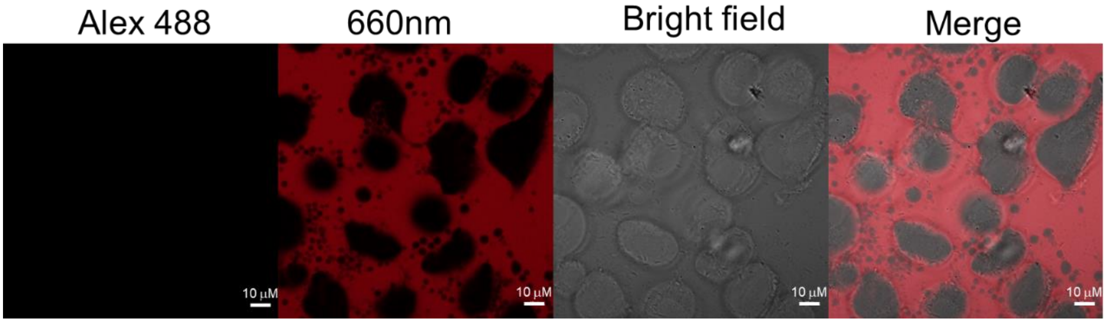

Supplementary Figure S17. Confocal microscopy images of HeLa cells incubated with the recombinant WSCP (150 μg/mL) for24 h. Scale bar: 10 μM. Images representative of *n* = 3.

1.13 Cellular uptake analysis by confocal microscope analysis.

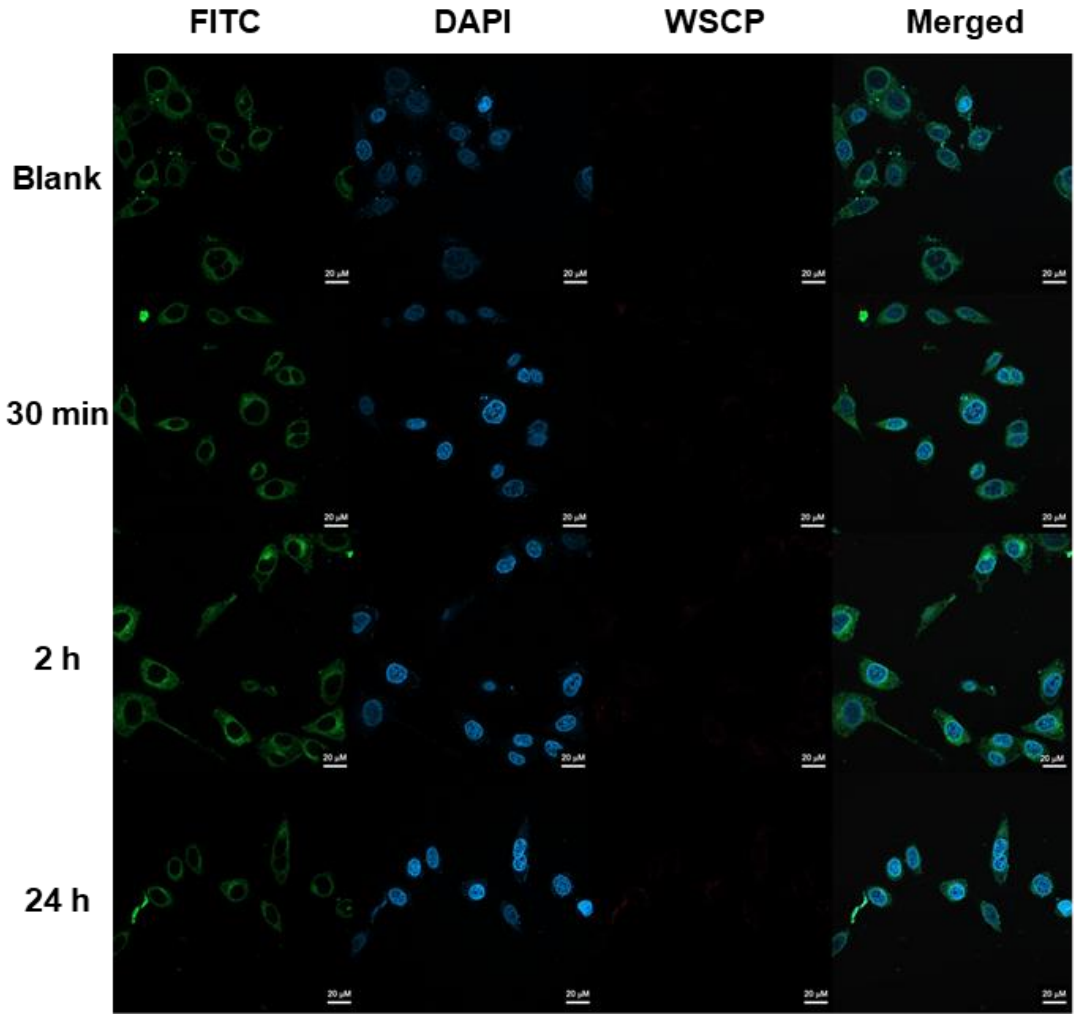

Supplementary Figure S18. Confocal microscopy images of normal liver cells (L02) after incubated with WSCP for different time. Scale bar: 20 μM. Images representative of $n = 2$.

1.14 Observation of unfixed HeLa cells with intracellular the recombinant WSCP through confocal microscope.

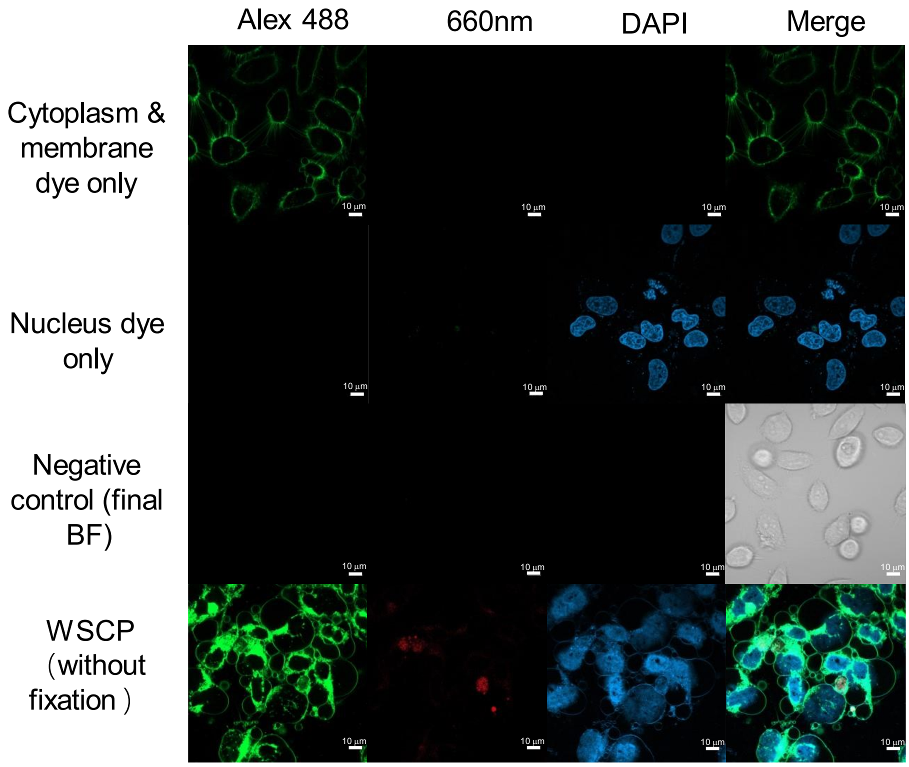

Supplementary Figure S19. Confocal microscopy images of unfixed HeLa cells incubated with or without the recombinant WSCP (150µg/mL) for 24 h upon irradiation of confocal microscope. Scale bar: 10 µM. Images representative of *n* = 3.

1.15 *In Vivo* Validation of Tumor Proliferation via Ki67 Immunofluorescent Staining in Tumor Sections

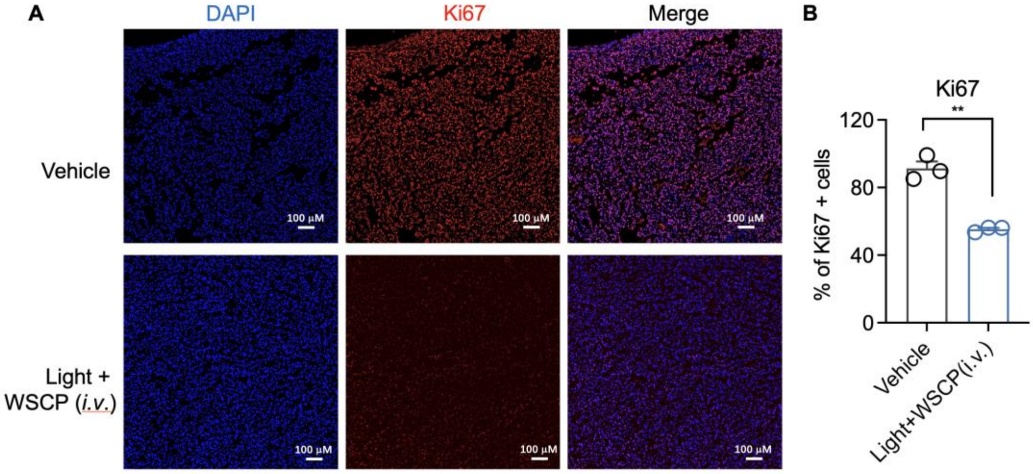

Supplementary Figure S20 Immunofluorescent staining of DAPI and Ki67. **A** Represent image of immunofluorescent staining of DAPI and Ki67. **B** Quantitative analysis of DAPI and Ki67 staining in tumor tissues. Images were taken at 10 × magnification. The results are expressed as the mean ± S.E.M. (*p < 0.05, **p < 0.01, ***p < 0.001).

1.16 HPLC data of WSCP (Purity determination)

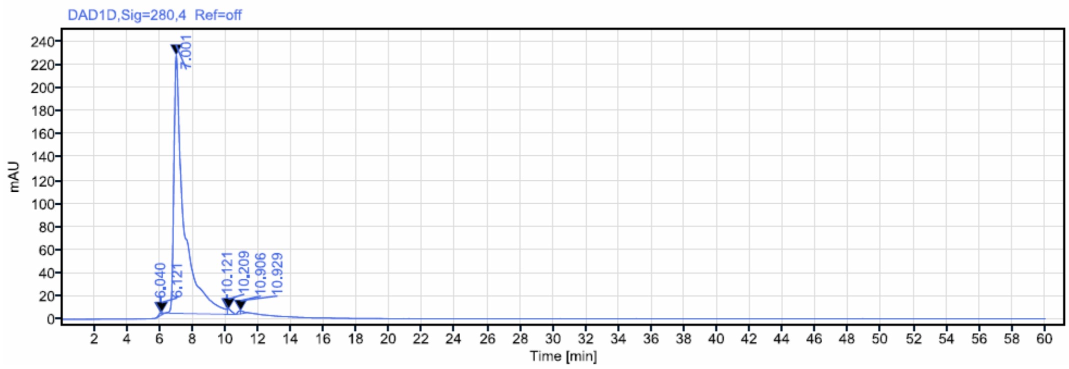

Signal: DAD1D,Sig=280,4 Ref=off

RT [min]	Type	Width [min]	Area	Height	Area%	Name
6.040	BV	0.34	26.10	2.22	0.27	
6.121	VV	0.14	8.94	1.77	0.09	
7.001	BV	3.82	9623.60	223.09	98.24	
10.121	VV	0.03	7.73	4.20	0.08	
10.209	VB	0.47	68.81	4.51	0.70	
10.906	BV	0.29	29.22	2.54	0.30	
10.929	VV	0.37	31.67	2.45	0.32	
		**Sum**	**9796.07**			

Supplementary Figure S20. HPLC analysis of WSCP at 280 nm.

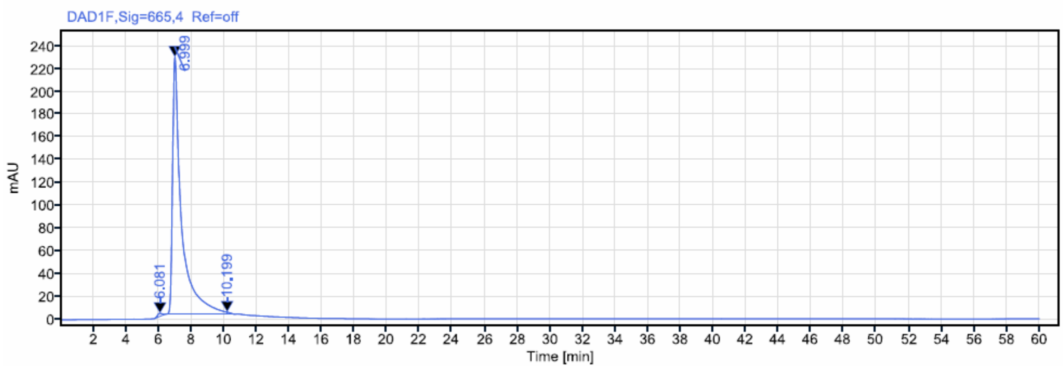

Signal: DAD1F,Sig=665,4 Ref=off

RT [min]	Type	Width [min]	Area	Height	Area%	Name
6.081	BV	0.57	49.75	2.64	0.57	
6.999	VV	3.77	8607.20	225.56	99.09	
10.199	VB	0.49	29.64	1.99	0.34	
		**Sum**	**8686.60**			

Supplementary Figure S21. HPLC analysis of WSCP at 665 nm.

2. References

[1]. Tian, Q.; Jiang, F.; Zou, R.; Liu, Q.; Chen, Z.; Zhu, M.; Yang, S.; Wang, J.; Wang, J.; Hu, J., Hydrophilic Cu9S5 nanocrystals: a photothermal agent with a 25.7% heat conversion efficiency for photothermal ablation of cancer cells in vivo. *ACS nano* **2011,** *5* (12), 9761-9771.
[2]. Xi, D.; Xiao, M.; Cao, J.; Zhao, L.; Xu, N.; Long, S.; Fan, J.; Shao, K.; Sun, W.; Yan, X., NIR light-driving barrier-free group rotation in nanoparticles with an 88.3% photothermal conversion efficiency for photothermal therapy. *Advanced Materials* **2020,** *32* (11), 1907855.
